# Optimising GPs’ communication of advice to facilitate patients’ self-care and prompt follow-up when the diagnosis is uncertain: a realist review of ‘safety-netting’ in primary care

**DOI:** 10.1136/bmjqs-2021-014529

**Published:** 2022-03-30

**Authors:** Claire Friedemann Smith, Hannah Lunn, Geoff Wong, Brian D Nicholson

**Affiliations:** 1 Nuffield Department of Primary Care Health Sciences, University of Oxford, Oxford, UK; 2 Chipping Surgery, Wotton-under-Edge, UK

**Keywords:** decision making, communication, patient safety, primary care, risk management

## Abstract

**Background:**

Safety-netting has become best practice when dealing with diagnostic uncertainty in primary care. Its use, however, is highly varied and a lack of evidence-based guidance on its communication could be harming its effectiveness and putting patient safety at risk.

**Objective:**

To use a realist review method to produce a programme theory of safety-netting, that is, advice and support provided to patients when diagnosis or prognosis is uncertain, in primary care.

**Methods:**

Five electronic databases, web searches, and grey literature were searched for studies assessing outcomes related to understanding and communicating safety-netting advice or risk communication, or the ability of patients to self-care and re-consult when appropriate. Characteristics of included documents were extracted into an Excel spreadsheet, and full texts uploaded into NVivo and coded. A random 10% sample was independently double -extracted and coded. Coded data wasere synthesised and itstheir ability to contribute an explanation for the contexts, mechanisms, or outcomes of effective safety-netting communication considered. Draft context, mechanism and outcome configurations (CMOCs) were written by the authors and reviewed by an expert panel of primary care professionals and patient representatives.

**Results:**

95 documents contributed to our CMOCs and programme theory. Effective safety-netting advice should be tailored to the patient and provide practical information for self-care and reconsultation. The importance of ensuring understanding and agreement with advice was highlighted, as was consideration of factors such as previous experiences with healthcare, the patient’s personal circumstances and the consultation setting. Safety-netting advice should be documented in sufficient detail to facilitate continuity of care.

**Conclusions:**

We present 15 recommendations to enhance communication of safety-netting advice and map these onto established consultation models. Effective safety-netting communication relies on understanding the information needs of the patient, barriers to acceptance and explanation of the reasons why the advice is being given. Reduced continuity of care, increasing multimorbidity and remote consultations represent threats to safety-netting communication.

**Key messages**

**What is already known on the topic?**

- Safety-netting was first formally described in 1987 and has since become best practice when dealing with diagnostic uncertainty in primary care as a means to support the patient to manage their symptoms when appropriate and reconsult when necessary. Its use, however, is highly varied and a lack of evidence-based guidance on its communication could be harming its effectiveness and putting patient safety at risk.

**What this study adds?**

- This study presents an evidence-based theory of and recommendations for how effective safety-netting might be achieved.

**How might this study affect research, practice or policy?**

- Effective safety-netting relies on general practitioner–patient understanding that should be built throughout the consultation and as such it should be incorporated into the whole of the consultation. We have highlighted a number of areas where further research is needed; not least what impact our recommendations will have on time-keeping within the consultation.

## Introduction

Diagnostic uncertainty is a defining feature of primary care with the majority of consultations ending without a definitive diagnosis.^1^ People attend primary care with undifferentiated symptoms and signs that could represent benign self-limiting illness or serious disease. Primary care clinicians in many jurisdictions must balance identifying serious illness with the need not to overwhelm specialist services or subject patients to unnecessary, costly and potentially harmful investigations.^2 3^ The ‘test of time’ is a useful consultation technique, allowing symptoms to develop or recede, or the suitability of treatment to become apparent. The test of time, however, risks harm to the patient if not used alongside safety-netting.

Safety-netting is regarded as best practice as a diagnostic strategy that involves monitoring patients with symptoms possibly indicative of serious illness until they are explained or resolved.^4 5^ The term in this context was first coined in 1987 by Roger Neighbour who described it as a back-up process for dealing with uncertainty in primary care whereby the general practitioner (GP) asks themselves three questions when making a preliminary diagnosis: ‘If I’m right, what do I expect to happen? How will I know if I am wrong? What would I do then?’^6^ It has been described by the National Institute for Health and Care Excellence (NICE) for England and Wales as ‘the provision of support for patients in whom the clinician has some uncertainty as to whether the patient has a self-limiting illness and is concerned that their condition may deteriorate’.^7^ Internationally, although the term is less widely used, the importance of a form of discharge or follow-up information is widely recognised.^8 9^ Safety-netting forms part of the assessment of new GPs,^10^ and clinical guidelines make reference to NICE’s safety-netting recommendations.^11^ Safety-netting is also used widely and has been observed in 65% and 90% of consultations in England and Scotland, respectively, alongside reports from GPs that they use it at the end of every consultation.^12–14^ As such, the opportunities for effective but also ineffective safety-netting are vast.

Research has documented varied understanding of safety-netting among GPs, varied use within consultations and inconsistent documentation of safety-netting in the clinical record.^12 15^ Safety-netting varies depending on the clinical strategies of the GP, the patient’s perceived ability to follow advice, the perceived risk of serious illness and in-consultation pressures.^12^ Patients do not recognise safety-netting as an established part of the consultation, lack understanding of what the clinician was trying to relay and can feel dismissed by it.^16^ The absence or incomplete provision of such information and advice also has implications for patient safety. Research exploring the reasons for delayed cancer diagnosis found that patients had felt dismissed in previous consultations when the GP had not provided an explanation of other possible causes for their symptom, or what to do next should the symptom persist.^17–21^ A false sense of security resulting from a failure to communicate the potential severity of the undiagnosed illness and the need for follow-up has been called ‘temporising’ in the US literature.^19 22^ A systematic review found many of the above factors to be barriers to patient engagement and highlighted safety-netting as a strategy through which these harms may be mitigated.^23^ This situation suggests that guidance on how safety-netting should be practised is needed. A number of UK-based organisations have created guidelines but these are mostly related to specific conditions and based on expert consensus.^24–27^ Crucially, as safety-netting is a widespread intervention for dealing with diagnostic *uncertainty*, the lack of guidance that can be applied across primary care settings and disease areas represents a significant knowledge and practice gap.

COVID-19 has introduced additional diagnostic uncertainty and complexity in communication by necessitating a large shift to remote consultation that is unlikely to be abandoned once the pandemic has abated.^28^ This has affected non-verbal communication and reduced opportunities for clinical examination and investigation.^29 30^ It is essential that we incorporate these lessons learnt from changes in clinical practice during the COVID-19 pandemic into safety-netting practice.

We conducted a realist review with the aim of providing information on how safety-netting may be effectively communicated to reduce the risks to patient safety outlined above. We did this with the input of an expert panel of professional and public volunteers who challenged and provided us with feedback and advice. The inclusion of stakeholder groups in research can improve the relevance of the topic, making outputs more valid and useful to user groups, and improve their implementation.^31 32^ The question that we refined and answered was: *How and why does safety-netting facilitate appropriate self-care and reconsultation, for whom and under what circumstances?* In answering this question using a realist review approach, we aimed to produce a programme theory of safety-netting communication that can be applied across primary care settings, communication mediums, patients groups and disease areas.

## Methods

Realist review is a theory-driven approach to evidence synthesis that uses relevant and trustworthy data to answer questions around what, why, how, when and for whom complex interventions work.^33^ A realist review methodology was chosen due to the complexity of safety-netting as an intervention, with the potential for variation at all stages from the provision of advice by the clinician to the interpretation and actioning of that advice by the patient. A benefit of realist review is its ability to produce a programme theory that can be transferred across contexts.^34^ Evidence-based context, mechanism and outcome configurations (CMOCs) are statements detailing the contexts in which certain mechanisms, that is, causal and often hidden processes, are triggered to bring about the specific outcomes of an intervention.^33^ A programme theory collates the individual CMOCs into an overall picture of how an intervention works.^35^

A protocol was registered with PROSPERO (CRD42019133194), we followed methods described by Pawson,^36^ and adhered to RAMESES quality and reporting standards^37 38^ (online supplemental appendix 1). We deviated from the protocol only in that we expanded the acceptable settings and participants from primary care settings and staff, to include any setting where discharge advice was being delivered and any staff involved, and acceptable interventions to include risk communication generally. Individuals receiving the advice included adult patients and adult carers or family members of patients unable to take responsibility for their own care.

10.1136/bmjqs-2021-014529.supp1 Supplementary data

**Figure 1:**
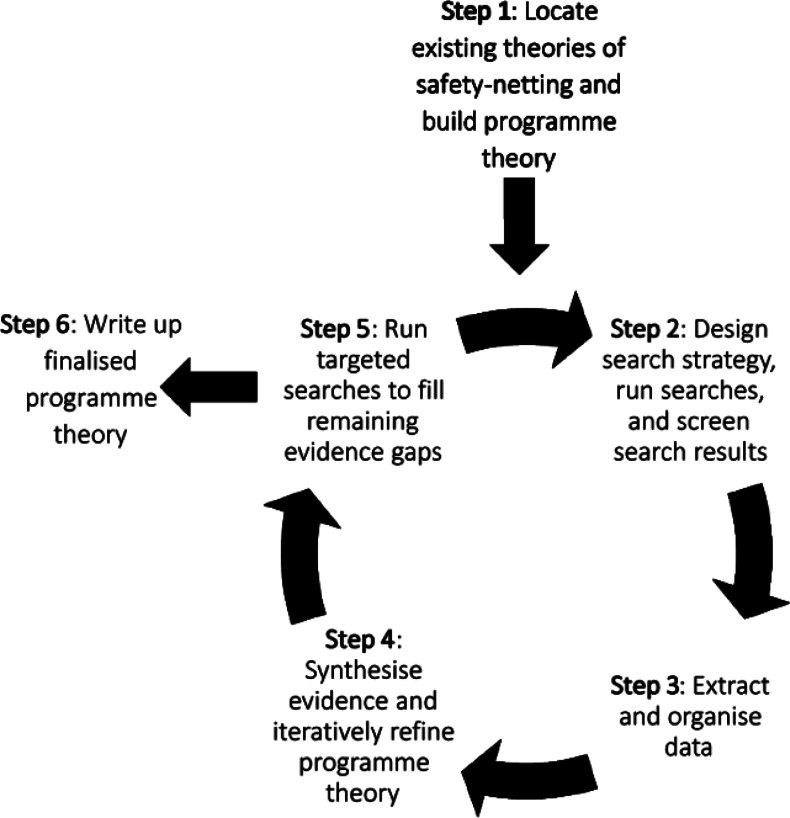
The realist review process.

We focused on the communication of safety-netting on the advice of our expert advisory panel (see below). This was to ensure the review would be feasible within the project timelines and because it was felt that the communication of safety-netting advice during the consultation was fundamental and further research could build on this work to examine its recording and follow-up. We carried out the review in six steps summarised in figure 1, described briefly below and in detail in online supplemental appendix 2.

10.1136/bmjqs-2021-014529.supp2 Supplementary data

An exploratory search was undertaken in PubMed and an initial programme theory developed and refined with the study team and expert panel (online supplemental appendix 3). Formal literature searches in five databases and Google were carried out in October 2019 (table 1). The searches were limited by publication date to papers published after 1987 as this was the year Neighbour first described safety-netting as we use the term today.^6^ Our Medline search strategy is available in online supplemental appendix 4. A title and abstract screen, followed by a full-text screen against the inclusion criteria (table 1), was carried out with a random 10% sample reviewed in duplicate to ensure consistency. Any discrepancies were resolved through discussion. The reference lists of all included articles were screened for relevant papers.

10.1136/bmjqs-2021-014529.supp3 Supplementary data

10.1136/bmjqs-2021-014529.supp4 Supplementary data

**Table 1:** The formal literature search

Intervention	Safety-netting advice given for symptoms where a diagnosis is not immediately apparent or illness is initially suspected to be self-limiting, risk communication.
Setting	Any healthcare setting where discharge advice is given, any setting where health risk is communicated, for example, public health messaging during a pandemic.
Participants	Any healthcare professional. Adult patients (≥18 years) of any gender, ethnicity or other demographic group. Adult carers or family members (≥18 years) of patients unable to take responsibility for their own care, for example, children or patients with developmental disorders, of any gender, ethnicity or other demographic group.
Study design	All study designs except case reports.
Outcome	Any outcome related to the understanding of the safety-netting advice or risk communication, or ability of the patient or carer to self-care when appropriate and reconsult when necessary.
Limits	1987–present. No limits on place or language of publication were used.
Databases searched	Medline, Embase, Health Management Information Consortium, Cumulative Index to Nursing and Allied Health Literature, PsychINFO. Targeted Google searches of charitable, professional and government bodies.

The characteristics of included materials were extracted into a preprepared Excel spreadsheet and the full texts uploaded into NVivo and coded, again with a random 10% consistency check. Papers were assessed for their relevance and rigour of the methods used. Judgements were made on the plausibility and coherence of the emerging programme theory.^39^ A description of each included study and its quality assessment is available in online supplemental appendix 5.

10.1136/bmjqs-2021-014529.supp5 Supplementary data

The coded data were synthesised and draft CMOCs were written. As the CMOCs were created and refined, we made judgements on how they related to each other, for example, whether it was necessary for one CMOC to precede another in the consultation. Following this process, we refined our initial programme theory into a realist programme theory (ie, one that contains realist causal explanations in the form of CMOCs). After each stage of evidence gathering and synthesis, we met with the expert panel to discuss the developing programme theory and CMOCs, identify missing information and refine the CMOCs. Targeted, iterative literature searches were carried out between June 2020 and April 2021 to update the search and provide information where gaps were identified. At our final expert panel meeting, the programme theory and CMOCs were finalised. On agreement of the final programme theory, the research process was written up in detail as described herein.

### Stakeholder and patient and public involvement

An expert panel of six primary care professionals and five patient volunteers was formed at the beginning of this study in 2019 and contributed until its completion in 2021. The panel was recruited through advertisements placed in Involvement Matters (https://www.clahrc-oxford.nihr.ac.uk/public-involvement/public-involvement-newsletters/involvement-matters), a bulletin of opportunities for members of the public to get involved in research, and in newsletters published by Oxfordshire Clinical Commissioning Group. The panel met four times, initially to help focus the review, and subsequently provided feedback and advice on the programme theory, CMOCs and our interpretation of the data, and on the dissemination plan.

## Results

### Document characteristics

**Figure 2:**
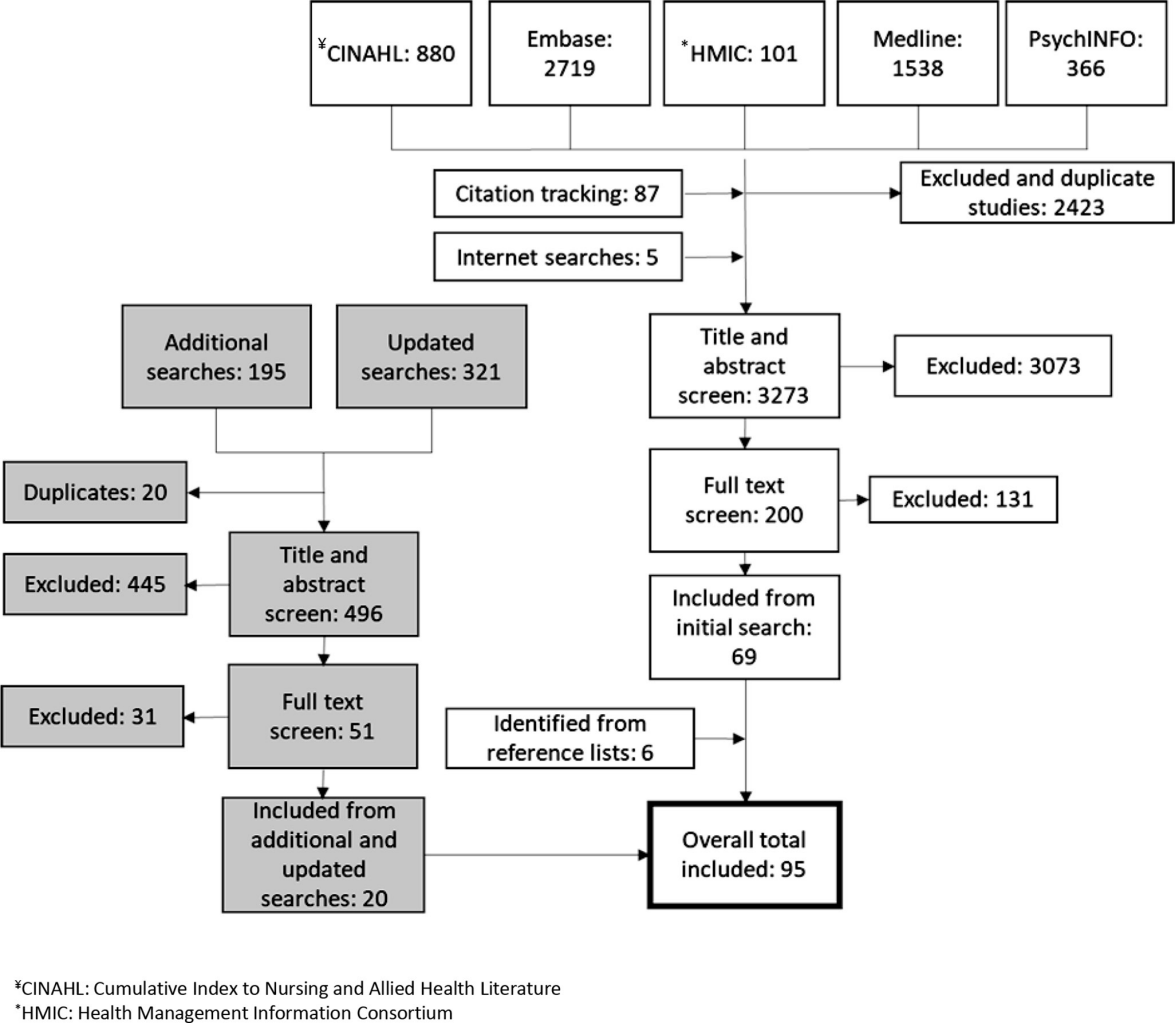
Flow of materials included in the review.

Ninety-five documents published between 1996 and 2021 from 10 countries (58 (61%) from the UK) were included (figure 2). The main reasons for exclusion were that materials discussed ‘safety net’ healthcare facilities for uninsured patients or did not contain information that could elucidate the context, mechanisms or outcomes of effective safety-netting advice. In the included documents, healthcare settings included in-hours GP care (43 (45%)); urgent, walk-in and out-of-hours care (16 (17%)); the community (5 (5%)); specialist or secondary care (8 (8%)); public healthcare (7 (7%)) and a mix of settings (16 (17%)). Included documents were research articles (64 (67%)), opinion pieces or commentaries (13 (14%)), web sources (9 (10%)), reports (4 (4%)), editorials or letters to editor (2 (2%)), clinical guidelines (2 (2%)) and books (1 (1%)). Of the research articles, 36 (56%) were qualitative studies, 8 (13%) were cross-sectional studies, 6 (10%) used mixed methods, 4 (6%) were literature reviews, 4 (6%) were systematic reviews, 4 (6%) were cohort studies and 2 (3%) were randomised trials.

### Nature of included data used to develop and support CMOCs and the programme theory

For each of the 95 included documents, global judgements were made (where possible) on: relevance of the data; appropriateness of methods used (if any) to support knowledge claims; plausibility of the findings and whether findings were supported by data in other documents. These global judgements were used to inform our judgement on the credibility of the explanations provided by the CMOCs we produced. Details of the included documents and our global judgements may be found in online supplemental appendix 5. The CMOCs alongside their explanatory credibility and supporting excerpts are presented in online supplemental appendix 6.

10.1136/bmjqs-2021-014529.supp6 Supplementary data

### Context, mechanism and outcome configurations

**Figure 3:**
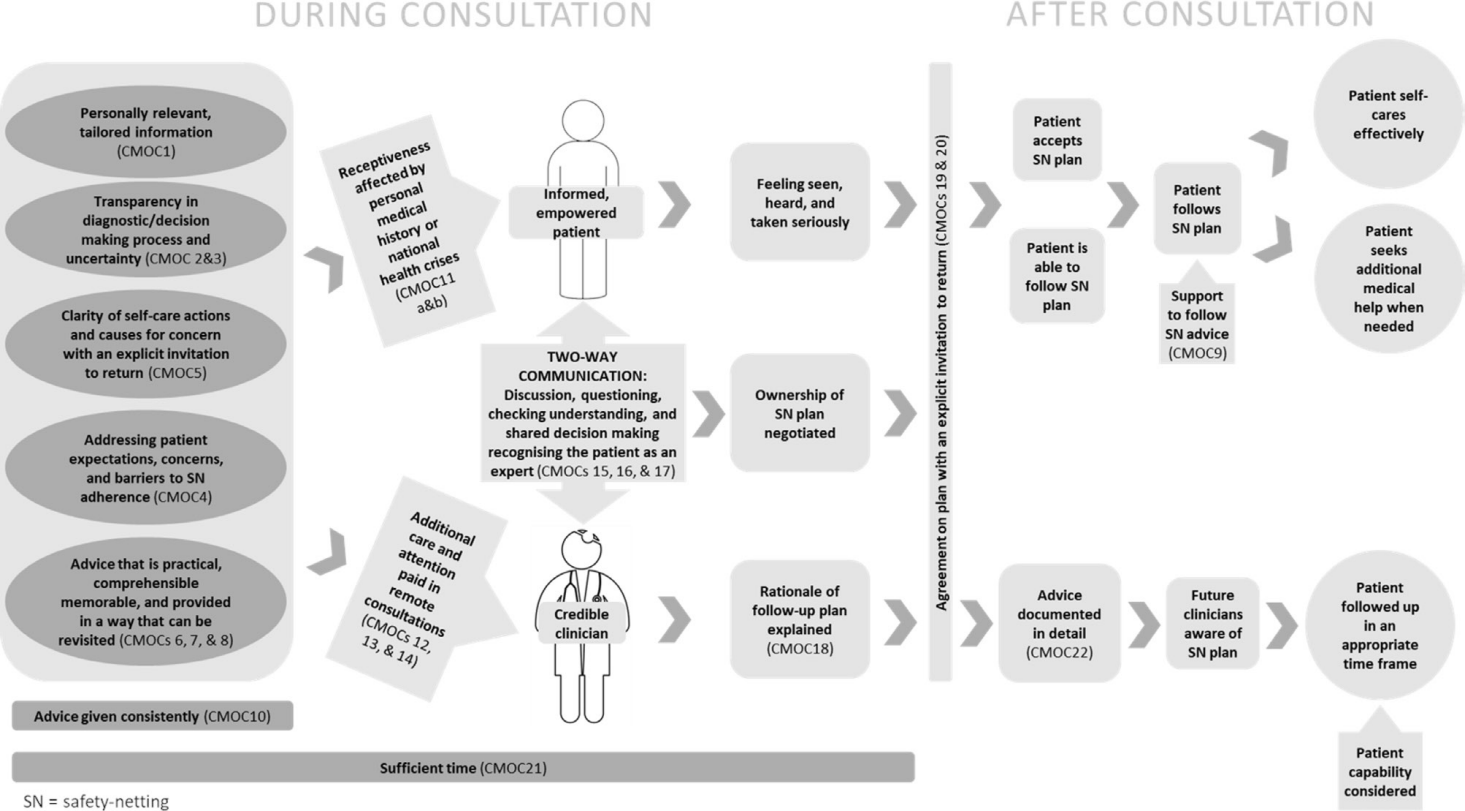
Programme theory of safety-netting. CMOC, context, mechanism and outcome configuration.

The CMOCs detailed in online supplemental appendix 6 contributed to the final programme theory of safety-netting which is presented in figure 3. The evidence relating to some CMOCs or some aspects of the CMOCs was limited. Where this is the case, it is indicated in the narrative.

### Safety-netting advice content

Providing patients with personally relevant information (CMOC1)^40–56^ that addresses their concerns and expectations (CMOC4)^27 40 49 53 57–74^ was important for them to accept and follow safety-netting advice as personalising information increased relevance and usefulness. Additionally, risk communicated based on the individual’s risk factors rather than population risk increased trust in the clinician giving the advice.^41^ This was especially important during a health crisis or pandemic where too much focus on, for example, risk related to ethnicity could be perceived as stigmatising.^41^

Explaining management plans (CMOC2),^16 50 62 75–87^ any diagnostic uncertainty (CMOC3),^4 16 25 42 46 55 62 64 75 87–91^ and allowing patients to ask questions led to greater transparency, improved understanding, and avoided false reassurance, thereby increasing confidence to reconsult. Explanations should include what safety-netting is and its purpose^87^ and the rationale for any actions taken (including no action).^84^ Research conducted in emergency departments supported this and added that this explanation should include why the patient is being discharged, which diseases were considered and the results of any tests.^74^ The need for the clinician to explain their diagnostic reasoning and logic behind their management strategy was particularly strong for parents or carers, whose tolerance of uncertainty was lower when consulting for someone under their care.^59 77^ When explaining uncertainty, research suggested that parents react more positively to uncertainty framed as most likely or differential diagnoses rather than explicit expressions of uncertainty.^91^

Appropriate reconsulting and self-care is facilitated if the clinician provides clarity about when the patient should be concerned (CMOC5)^4 12 16 25 27 47 49 50 53 55–57 59 64 66 72 75–77 79 82 83 88 89 92–104^ and practical advice is offered by the clinician (CMOC6),^4 16 27 44 47 49 53 55 57 58 60 68–70 74 77–79 89 93 97 100 105 106^ as the patients’ sense of control and confidence in their own abilities is increased. Advice for managing symptoms, when to suspect treatment failure and concerning time frames were all important.^53 83 89 92 94 97^ Assessing and recognising problematic symptoms was particularly important for situations where a parent or carer is making the decision of when to seek help.^72 77 93^ Demonstrating signs and tests, for example, the tumbler test for meningococcal disease provided more clarity on what to look for than verbal or written information alone.^49 56 98^

Safety-netting advice should be comprehensible (CMOC7),^49 62 65 77 83 95 102 107^ memorable (CMOC8),^14 57^ accompanied by materials allowing the patient to revisit it (CMOC9),^26 27 47 49 53 55–57 59 62 63 65 77 88 89 98–100 103 107–112^ and given consistently when there is uncertainty irrespective of the perceived risk of the clinical presentation (CMOC10).^27 63^ Avoiding jargon, abbreviations and using language that could be easily understood were important but patients did not want their clinician to omit technical information that could facilitate understanding.^77^ For patients who are less able to understand written information, using audio-visuals, illustrations and colour coding helped to improve understanding.^49 77^ Strategies like ‘chunking’ and ordering verbal information according to priority were suggested^14 57^ but providing materials that could be referred back to remove the need to memorise information. This was also true for telephone consultations and the clinician should have the ability to email advice leaflets or give the patient a website where they can access advice.^53^ Information was deemed trustworthy if it was endorsed by organisations patients considered reputable (such as the National Health Service (NHS) in the UK) and this prevented internet searches that raised anxiety.^49 100^ However, written safety-netting advice was not considered a substitute for verbal discussion.^56 99^

### Additional considerations

Patients were more likely to act on safety-netting advice if the clinician integrated their wider knowledge of the patient and addressed potential sources of anxiety (CMOC11a).^41 42 62 73 100 102 113–120^ Characteristics such as young age, being a first time or single parent, previous missed diagnoses, traumatic experiences with the healthcare system and alarming symptoms in children are potential causes of anxiety to address.^62 100 102 119 120^ This was especially important when the patient was not known to the clinician.^62^

The COVID-19 pandemic highlighted additional factors that could impede patients’ responsiveness to safety-netting advice. When the clinician shows that they are aware of and addresses concerns around a pandemic or ongoing health crisis, the patient is better equipped to assess how the safety-netting advice impacts their own risk, and so may view it as more actionable (CMOC11b).^41 42 102 113–119 121^ Patients were more responsive if the risks of the illness being safety-netted were balanced against the risks of the pandemic,^121^ the patient was informed of any disruption to services,^121^ and follow-up consultations were pre-arranged with patients who were fearful or reticent to ‘bother’ the doctor.^113^ Providing clear, detailed information was vital at a time when patients may be more easily confused,^42^ exposed to misinformation, and experiencing increased fear and anxiety.^114 118^ Patients should also be informed that they can change their mind if they decline investigations being offered.^121^

Recent documents explored communication during remote consultations. Important aspects to compensate for the impact of reduced non-verbal communication (CMOC12)^60 65 87 122^ included ensuring careful listening and questioning,^60 122^ and actively seeking feedback on whether information was understood.^87^ The literature contributing to CMOCs 13 and 14 was limited and only of moderate relevance to this review but suggested that patient satisfaction with the consultation and information transfer was facilitated by factors such as minimised distractions, good lighting and attention to the screen.^122 123^ These CMOCs were discussed with our expert panel who felt they were important to include.

### Agreeing the plan

The safety-netting plan should be made through discussion with the patient (CMOC15).^55 70 109 124^ This made the patient feel they had been taken seriously and ensured that the plan was manageable.^109 124^ The expert panel further highlighted that ownership of the plan should be negotiated to avoid misunderstandings about who held the responsibility for follow-up, and that it be made clear how the safety-netting plan could change if the patient’s situation evolves, for example, if new symptoms develop.

When giving safety-netting advice, the clinician should acknowledge the personal knowledge of the patient/parent/carer of their own or their charge’s health (CMOC17).^93 106 125^ Personal knowledge can include knowledge of their own body and of the usual behaviour of the person in their care.^125^ Recognition of this expertise reduced the feeling of hierarchy between patient and clinician, reduced anxiety and increased confidence to reconsult.^59 93^

The rationale for the follow-up plan should be explained (CMOC18)^4 16 55 70 71 126^ and the plan should be agreed between the clinician and patient (CMOC19)^12 16 25 50 51 55 85 92 106 109 124 127 128^ so the expectations for follow-up are explicit, any misunderstandings are reduced and the patient is followed up appropriately. That the clinician should check the patient’s understanding of the safety-netting advice to reduce misunderstandings and promote appropriate reconsulting was supported by many data sources (CMOC16).^14 25 47 48 53 56 64 70 86 95 97 102 121 124^ Again, our expert panel suggested that checking the patient understands that the advice may change as their illness evolves be included. Checking understanding is especially important when the consultation is not face-to-face. The literature highlighted the importance of active elicitation of patient questions as some patients will not initiate questioning.^129^

When the clinician explicitly invites the patient to return, even for the same symptoms, the patient is supported to seek further medical advice (CMOC20).^16 45 60 93 98 106 109 112^ Receiving self-care advice could present a barrier to reconsultation and patients feared being labelled as time wasters if they came back with the same symptoms^93^ and so the explicit invitation to return may legitimise a reconsultation.^45 60^

Finally, when sufficient time was allowed, safety-netting advice could be more fully explained, understood and discussed (CMOC21).^47 54^ Although this CMOC had only a small number of documents supporting it, these were highly relevant and the findings that safety-netting under pressure can lead to it being delivered carelessly^47^ were supported by our expert panel.

### Documenting safety-netting

A range of documents supported CMOC22.^25 26 47 50 53 56 89 94 97 99 102 104 121 127^ that when safety-netting advice is documented in sufficient detail in the patient’s record, clinicians caring for the patient in the future are aware of what has been discussed and decided, so can continue care effectively. The data did not suggest that every detail should be recorded but rather that it should be sufficient for continuity of care.^26 127^ Thorough recording of safety-netting advice was also highlighted as important from a medico-legal perspective.^56^

## Discussion

This realist review sets out the contexts of effective communication of safety-netting advice, the mechanisms that the contexts trigger, and the outcomes of adherence, self-care, and timely reconsultation or follow-up. We have drawn on the safety-netting, risk communication and care management literature to build a programme theory that has been extensively discussed and agreed by an expert panel of professional and public volunteers. Our findings can be divided into four domains: safety-netting advice content, additional considerations, agreeing the plan and documenting safety-netting. The thread running through these domains is that patients are more likely to follow safety-netting advice if they understand what safety-netting is, why it is being used, what the safety-netting actions are and who holds responsibility for the safety-netting actions. Based on our findings, we present recommendations for the effective communication of safety-netting in table 2.

**Table 2:** Recommendations for practice and illustrative links to established consultation models

Recommendations for clinicians using safety-netting*†	Stages of consultation models relating to recommendation		
	Pendleton *et al* 150	Calgary-Cambridge^151^	Neighbour’s checkpoints^6^
1. Consider providing safety-netting advice to all patients where there is uncertainty in the diagnosis or the potential for the diagnosis to evolve.	-	-	-
2. Offer safety-netting advice in simple terms and tailor it to the patient’s presentation. Do not omit technical information that may improve understanding.	Task 4 (shared understanding)	Step 4 (explanation and planning)	Safety-netting
3. Offer patients the opportunity to discuss their expectations and concerns and ensure they are addressed in the safety-netting advice.	Task 1 (define reason)	Steps 1 and 2 (initiating the session and gathering information)	Summarising
4. Offer an initial diagnosis and describe the expected natural history with practical instructions for self-care and specific situations that should be cause for concern in the safety-netting advice.	Task 4 (shared understanding)	Step 4 (explanation and planning)	Handover
5. Offer resources that will allow the patient to revisit safety-netting information in their own time.	Task 6 (use time and resources appropriately)	Step 4 (explanation and planning)	Safety-netting
6. Consider using techniques such as ‘chunking’ to improve recall of the safety-netting information.	Task 4 (shared understanding)	Step 4 (explanation and planning)	Safety-netting
7. Offer a safety-netting plan that is sensitive to and addresses factors that may make the patient less receptive to safety-netting advice.	Task 3 (choose appropriate action with patient)	Steps 2 and 4 (gathering information and explanation and planning)	Connecting
8. Offer the patient the opportunity to discuss and share in the decision-making of the safety-netting plan.	Tasks 3 and 5 (choose appropriate action with patient and involve the patient)	Step 4 (explanation and planning)	Handover
9. Offer an explanation for the specific safety-netting plan and follow-up plan, and include a discussion of any uncertainty in the initial diagnosis.	Task 5 (involve the patient)	Step 4 (explanation and planning)	Handover
10. Consider actively checking that the patient understands the safety-netting plan.	Task 4 (shared understanding)	Steps 4 and 5 (explanation and planning, closing the session)	Handover
11. Consider explicitly acknowledging the patient’s greater knowledge and ability to make judgements about their own health.	Task 5 (involve the patient)	Step 4 (explanation and planning)	Handover
12. Offer the patient an opportunity to explicitly agree to the follow-up plan.	Task 5 (involve the patient)	Step 4 (explanation and planning)	Handover
13. Offer the patient an explicit invitation to return for further medical advice, even if it is for the same symptom(s).	Task 4 (shared understanding)	Step 5 (closing the session)	Safety-netting
14. Consider building in elements of safety-netting throughout the consultation to avoid it being rushed at the end of the consultation.	All	All	All
15. Offer sufficient detail about the safety-netting advice in the patient’s medical record that future clinicians are able to understand what care was given and continue it appropriately.	-	-	-

*Recommendations are worded as per the NICE wording convention where ‘offer’ signifies high explanatory credibility of the recommendation and ‘consider’ signifies moderate explanatory credibility.^152^

†Findings where the explanatory credibility of the CMOC was rated at low are not included in these recommendations.

CMOC, context, mechanism and outcome configuration; NICE, National Institute for Health and Care Excellence.

### Strengths and limitations

The major strength of this review lies in the range of materials used to build the CMOCs and the final programme theory. To date, the literature on safety-netting has been dominated by commentaries and although qualitative, observational and experimental research is starting to emerge, this still makes up a minority of the literature. Additionally, this review is strengthened by the inclusion of an expert panel of professional and public volunteers. This expert panel was involved for the duration of the study, and they have discussed each of the CMOCs and the final programme theory from the perspective of the individual providing, and the individual receiving the safety-netting advice. Finally, the included literature covers a wide range of disease areas and so our recommendations are not restricted to specific illnesses, which is a strength given safety-netting is most often used in the absence of a firm diagnosis.

Our aim was to create a programme theory that could be applied to all disease areas, patient groups, communication mediums and primary care settings. We aimed to make the output of this research applicable in all primary care settings including out of hours, urgent care, and pharmacy as the lack of continuity of care and reduced access to patient records suggests that careful safety-netting may be of even greater importance in these settings. However, most of the literature retrieved was linked to in-hours primary care meaning our findings should be applied with caution to other settings. Additionally, there was only a small amount of literature available for CMOCs for some communication mediums. As all of the CMOCs were discussed and agreed by our expert panel, we have included all CMOCs in our programme theory, highlighting areas for future research. We were unable to make recommendations specifically tailored to the communication of safety-netting during remote consultations due to the lack of data which weakened the explanatory credibility of a small number of the CMOCs. Although many of our recommendations will apply to remote consultations, future research should explore whether patient understanding of and adherence to safety-netting advice is affected by remote consultations and what measures should be taken to facilitate safety-netting communication. The included literature reported findings relevant to a range of groups, for example, parents, carers and patients with limited literacy. Of patient factors, ethnicity was the least well explored. While we do not urge the same caution in applying our recommendations across patient groups, we strongly advise that future safety-netting research specifically investigates the effect that ethnicity, cultural attitudes towards health and healthcare, and GPs’ cultural competence^130^ may have on the effectiveness of safety-netting advice.

### Links to existing research

This review highlights both relational and informational continuity of care as important for effective safety-netting. This is supported in the literature examining the effects of continuity of care in that greater continuity has been linked to decreased use of out-of-hours services, acute hospital admissions and mortality.^131 132^ The reasons proposed for this effect mirror the mechanisms reported herein, in that greater continuity is suggested to lead to greater patient trust, better communication and so greater adherence to medical advice.^131 133^

Safety-netting shares commonalities with the personalised care planning, shared decision-making, risk communication and communications training literature.^134–137^ The safety-netting literature reflects that of shared decision-making in that both emphasise the importance of addressing the information needs of the patient and that the patient is given the opportunity to question the management plan.^138^ More collaborative styles of consulting are, however, likely to have implications for timekeeping and clinicians are reported to be less likely to engage with shared decision-making if they perceive it as an additional demand on their time.^139 140^ The literature reports an average increase of 2.6 min in the length of consultations that include shared decision-making.^141^ Although this increase is reportedly not *statistically significant,* the cumulative effect of even small increases could make safety-netting infeasible for many.^142 143^

It is likely that the extent to which safety-netting is integrated into the consultation will impact its feasibility. Table 2 maps our safety-netting recommendations onto three primary care consultation models selected for their popularity and relative patient-centeredness (Pendleton *et al*, Calgary-Cambridge and Neighbour^144^), to demonstrate where safety-netting actions overlap with or are integrated into the ‘model’ consultation. While taught consultation models and clinical practice may markedly differ, and often do, this mapping provides an indication that safety-netting should not be thought of as an additional task but rather the result and summation of existing recognised components of the consultation. Only two of our recommendations did not map to all consultation models. These recommendations concerned the communication of safety-netting advice in all cases of diagnostic uncertainty and the documentation of advice in the record. These aspects of communication may become integrated into future iterations of consultation models based on the findings of this review.^145^

### Implications for practice and research

Conceptualising safety-netting as something that happens in the last 30 seconds of the consultation runs counter to our findings. The safety-net should be considered the product of a shared understanding between the doctor and patient that develops throughout the consultation and which is supported by in-depth knowledge of patients built by GPs over time. Lack of time is given as a reason why safety-netting is often poorly practised,^12 23^ and continuity of care is declining in primary care.^146^ Research is required to understand the impact of integrating these recommendations on consultation length and the amount of additional time that is likely to be required, and to establish how clinician–patient relationships can be fostered by safety-netting systems in circumstances where continuity of care is limited.

We found no materials which included advice for safety-netting patients with multiple issues. This is important as the average number of issues dealt with per consultation is reported to be 2.5,^147^ and likely to increase as the consulting population ages. A recent study found that when multiple issues are raised during the consultation, the likelihood of GPs providing safety-netting advice and recording advice in the patient’s record decreased with each additional issue.^15^ Addressing safety-netting in the context of multimorbidity should be a priority for future research.

Our review focused on the communication of safety-netting advice within the consultation. Future research should investigate how follow-up of safety-netting advice is best implemented. Often clinicians prefer the responsibility of follow-up to rest with the patient (so long as they are deemed able) and that while some patients accept this (so long as they have been given enough information), other patients prefer more active follow-up.^16 55^ What effective follow-up looks like, and whether there is a role to play for electronic safety-netting solutions should be established.^148 149^

Finally, training and continuing professional development of primary care clinicians might be updated to include these findings. Changes in patient demographics and illness profiles, the use of technology in the consultation, and workforce pressures mean that the practice and importance of safety-netting will continue to evolve. It is important that training and research keep pace with this.

## Conclusion

We present a theory and set of recommendations for effective safety-netting communication but acknowledge that at first glance, these may seem daunting in an already crowded consultation, of which safety-netting is usually considered only a small part. Patients are more likely to follow safety-netting advice if they understand what safety-netting is, why it is being used, what the safety-netting actions are and who holds responsibility for safety-netting actions. We propose that these elements of effective safety-netting, with few exceptions, are already incorporated into the ‘model’ consultation.

## Data Availability

All data relevant to the study are included in the article or uploaded as supplemental information. Not applicable.
