# Supplementary material for: Optimising GPs’ communication of advice to facilitate patients’ self-care and prompt follow-up when the diagnosis is uncertain: a realist review of ‘safety-netting’ in primary care
Source: BMJ Qual Saf. 2022 Mar 30;31(7):541–54. doi: 10.1136/bmjqs-2021-014529 (PMC9234415; doi:10.1136/bmjqs-2021-014529)
Supplement: Supplementary data [file bmjqs-2021-014529supp001.pdf]

Appendix 1: RAMESES Publication Standards Checklist

**TITLE**			**PAGE NUMBER**
1		In the title, identify the document as a realist synthesis or review	1
ABSTRACT			
2		While acknowledging publication requirements and house style, abstracts should ideally contain brief details of: the study's background, review question or objectives; search strategy; methods of selection, appraisal, analysis and synthesis of sources; main results; and implications for practice.	3
INTRODUCTION			
3	Rationale for review	Explain why the review is needed and what it is likely to contribute to existing understanding of the topic area.	4
4	Objectives and focus of review	State the objective(s) of the review and/or the review question(s). Define and provide a rationale for the focus of the review.	4
METHODS			
5	Changes in the review process	Any changes made to the review process that was initially planned should be briefly described and justified.	5
6	Rationale for using realist	Explain why realist synthesis was considered the most appropriate method to use.	5

**TITLE**			**PAGE NUMBER**
	synthesis		
7	Scoping the literature	Describe and justify the initial process of exploratory scoping of the literature.	6
8	Searching processes	While considering specific requirements of the journal or other publication outlet, state and provide a rationale for how the iterative searching was done. Provide details on all the sources accessed for information in the review. Where searching in electronic databases has taken place, the details should include, for example, name of database, search terms, dates of coverage and date last searched. If individuals familiar with the relevant literature and/or topic area were contacted, indicate how they were identified and selected.	6
9	Selection and appraisal of documents	Explain how judgements were made about including and excluding data from documents, and justify these.	6&7
10	Data extraction	Describe and explain which data or information were extracted from the included documents and justify this selection.	7
11	Analysis and synthesis processes	Describe the analysis and synthesis processes in detail. This section should include information on the constructs analyzed and describe the analytic process.	7
RESULTS			
12	Document flow	Provide details on the number of documents assessed for eligibility and included in the review with reasons for	8

**TITLE**			**PAGE NUMBER**
	diagram	exclusion at each stage as well as an indication of their source of origin (for example, from searching databases, reference lists and so on). You may consider using the example templates (which are likely to need modification to suit the data) that are provided.	
13	Document characteristics	Provide information on the characteristics of the documents included in the review.	9
14	Main findings	Present the key findings with a specific focus on theory building and testing.	15-17
DISCUSSION			
15	Summary of findings	Summarize the main findings, taking into account the review's objective(s), research question(s), focus and intended audience(s).	18
16	Strengths, limitations and future research directions	Discuss both the strengths of the review and its limitations. These should include (but need not be restricted to) (a) consideration of all the steps in the review process and (b) comment on the overall strength of evidence supporting the explanatory insights which emerged.<br>The limitations identified may point to areas where further work is needed.	18
17	Comparison with existing literature	Where applicable, compare and contrast the review's findings with the existing literature (for example, other reviews) on the same topic.	18&19
18	Conclusion and recommendations	List the main implications of the findings and place these in the context of other relevant literature. If appropriate, offer recommendations for policy and practice.	19&20

TITLE			PAGE NUMBER
19	Funding	Provide details of funding source (if any) for the review, the role played by the funder (if any) and any conflicts of interests of the reviewers.	23

Wong, G., Greenhalgh, T., Westhorp, G. et al. RAMESES publication standards: realist syntheses. *BMC Med* 11, 21 (2013). https://doi.org/10.1186/1741-7015-11-21
