# Supplementary material for: Optimising GPs’ communication of advice to facilitate patients’ self-care and prompt follow-up when the diagnosis is uncertain: a realist review of ‘safety-netting’ in primary care
Source: BMJ Qual Saf. 2022 Mar 30;31(7):541–54. doi: 10.1136/bmjqs-2021-014529 (PMC9234415; doi:10.1136/bmjqs-2021-014529)
Supplement: Supplementary data [file bmjqs-2021-014529supp002.pdf]

Appendix 2: Detailed description of the Realist Review method used

Step 1: Locate existing theories and build initial programme theory

[Figure 1 about here]

An exploratory search was undertaken in PubMed to locate existing theories of safety-netting and the gathered literature was discussed by the study team. The initial programme theory was developed by CFS, and shared with the research team for comment and feedback. This process was repeated until the study team agreed on the draft initial programme theory, at which point it was discussed with the expert panel at a face-to-face meeting. Amendments were made following this meeting, and the initial programme theory is available in Appendix 3.

Step 2: Run formal searches and screen results

A formal literature search was piloted, refined, and then carried out with the assistance of a librarian using key words identified in our exploratory search. Medline, Embase, Health Management Information Consortium (HMIC), Cumulative Index to Nursing and Allied Health Literature (CINAHL), PsychInfo, as well as targeted Google searches of charitable, professional, and government bodies were searched from 1987 to October 2019. We limited our searches to papers published from 1987 onwards as this was the year that Neighbour first described safety-netting as we use the term today (6). Our Medline search strategy is available in Appendix 4**,** searches in other databases used the same terms but were adapted to each database. Relevant documents were identified through a title and abstract screen, followed by a full text screen against the inclusion criteria (Table 1) by CFS with a random 10% sample of materials reviewed in duplicate by either BDN or HL to ensure consistency. Any discrepancies were resolved through discussion. The reference lists of all included articles were also screened for potentially relevant papers.

Intervention	Safety-netting advice given for symptoms where a diagnosis is not immediately apparent or illness is initially suspected to be self-limiting, risk communication.
Setting	Any healthcare setting where discharge advice is given, any setting where health risk is communicated, for example public health messaging during a pandemic.
Participants	Any healthcare professional.<br>Adult patients (≥18 years) of any gender, ethnicity, or other demographic group.<br>Adult carers or family members (≥18 years) of patients unable to take responsibility for their own care, for example, children or patients with developmental disorders, of any gender, ethnicity, or other demographic group.
Study design	All study designs except case reports
Outcome	Any outcome related to the understanding of the safety-netting advice or risk communication, or ability of the patient or carer to self-care when appropriate and re-consult when necessary.
Limits	1987 – present. No limits on place or language of publication were used.

Table 1. Inclusion criteria

Step 3: Extract data

The characteristics of included materials were extracted into a pre-prepared Excel spreadsheet. The full texts of included materials were uploaded into NVivo and coded by CFS, with a random 10% consistency check carried out by HL. At this stage, the inclusion of papers was based on the relevance of the paper (whether the paper contained data that was judged to be relevant to the programme theory), and the rigour of the methods used (where possible). Judgements were also made of the

plausibiliy and coherence of the emerging programme theory (40). A description of each included study and its quality assessment can be found in Appendix 5.

Step 4: Synthesise evidence and refine programme theory

The coded data was synthesised and the outcomes of safety-netting considered. This was done by considering what the data described as the important outcomes of safety-netting, what conditions or actions as part of safety-netting were needed to bring about those outcomes (contexts), whether any explanations were given or could be inferred for what caused the outcome (mechanisms). Additionally, relevant data was drawn from across the included documents to develop and refine the draft CMOCs so that they were able to provide causal explanations for different settings and patient groups. Draft CMOCs were written by CFS and discussed and refined with GW and BDN in the first instance. As the CMOCs were created and refined, we made judgements, based on relevant data from within included materials, on how the CMOCs related to each other, for example, whether it was necessary for one CMOC to precede another in the consultation. Following this process we refined our initial programme theory into a realist proramme theory (i.e. one contains realist causal explanations in the form of CMOCs). After each stage of evidence synthesis following the formal search and the subsequent targeted searches (see step 5) we met either face-to-face or virtually with an expert panel made up of primary care professionals and patient representatives. At each meeting the developing programme theory and CMOCs were discussed, missing information identified, and (where needed) CMOCs refined based on their feedback and advice.

Step 5: Targeted searches

Targeted, iterative literature searches were carried out between June 2020 and April 2021 following the discussion of the programme theory and CMOCs with the research team and expert panel. The aim of these searches was to update our initial search and provide information where gaps were identified. The literature found through these searches was screened, data extracted, and synthsised as described above. For example, in the early stages of the COVID-19 pandemic it was decided that safety-netting during remote consultations and health crises was important to include but insufficient data on these topics had been found. As such, targeted searches on safety-netting when the consultation is not face-to-face, and health risk communication during health crises or pandemics were carried out. At our final expert panel meeting, the programme theory and CMOCs were agreed and finalised.

Step 6: Write up finalised programme theory

On agreement of the final programme theory the research process was written up in detail as described herein.
