# Supplementary material for: Optimising GPs’ communication of advice to facilitate patients’ self-care and prompt follow-up when the diagnosis is uncertain: a realist review of ‘safety-netting’ in primary care
Source: BMJ Qual Saf. 2022 Mar 30;31(7):541–54. doi: 10.1136/bmjqs-2021-014529 (PMC9234415; doi:10.1136/bmjqs-2021-014529)
Supplement: Supplementary data [file bmjqs-2021-014529supp004.pdf]

Appendix 4: Medline search strategy

# ▲	Searches
1	safety netting.mp.
2	safety net*.ti,ab.
3	(((talk* or communicat* or explain* or decision making) adj5 (uncertain* or risk* or concern*)) and (re-consult* or reconsult* or re-assess* or reassess* or plan* or referral* or escalat*)).ti,ab.
4	(((talk* or communicat* or explain* or decision making) adj5 (uncertain* or risk* or concern*)) and ((follow up or future or further or additional or extra or seek*) adj5 (appointment* or consult* or help or advice))).ti,ab.
5	(((talk* or communicat* or explain* or decision making) adj5 (uncertain* or risk* or concern*)) and patient safety).ti,ab.
6	(((talk* or communicat* or explain* or decision making) adj5 (uncertain* or risk* or concern*)) and (information adj3 need*)).ti,ab.
7	((diagnos* adj3 uncertain*) and (re-consult* or reconsult* or re-assess* or reassess* or plan* or referral* or escalat* or return*)).ti,ab.
8	((diagnos* adj3 uncertain*) and ((follow up or future or further or additional or extra or seek*) adj5 (appointment* or consult* or help or advice))).ti,ab.
9	((diagnos* adj3 uncertain*) and patient safety).ti,ab.
10	((diagnos adj3 uncertain*) and (information adj3 need*)).ti,ab.
11	(red flag* and (re-consult* or reconsult* or re-assess* or reassess* or plan* or referral* or escalat* or return*)).ti,ab.
12	(red flag* and ((follow up or future or further or additional or extra or seek*) adj5 (appointment* or consult* or help or advice))).ti,ab.
13	(red flag* and patient safety).ti,ab.
14	(red flag* and (information adj3 need*)).ti,ab.
15	(((worr* or concern*) adj3 (symptom* or sign* or feature*)) and (re-consult* or reconsult* or re-assess* or reassess* or plan* or referral* or escalat* or return*)).ti,ab.
16	(((worr* or concern*) adj3 (symptom* or sign* or feature*)) and ((follow up or future or further or additional or extra or seek*) adj5 (appointment* or consult* or help or advice))).ti,ab.
17	(((worr* or concern*) adj3 (symptom* or sign* or feature*)) and patient safety).ti,ab.
18	(((worr* or concern*) adj3 (symptom* or sign* or feature*)) and (information adj3 need*)).ti,ab.
19	2 or 3 or 4 or 5 or 6 or 7 or 8 or 9 or 10 or 11 or 12 or 13 or 14 or 15 or 16 or 17 or 18
20	Ambulatory Care/
21	exp Ambulatory Care Facilities/
22	general practice/ or family practice/
23	general practitioners/ or physicians, family/ or physicians, primary care/
24	Primary Health Care/
25	Office Visits/
26	exp Emergency Service, Hospital/
27	Emergency Medical Services/
28	(ambulatory adj3 (care or setting? or facilit* or ward? or department? or service?)).ti,ab.
29	((general or family) adj2 (practi* or physician? or doctor?)).ti,ab.
30	(primary care or primary health care or primary healthcare).ti,ab.

31	(community adj3 (practitioner? or nurse? or pharmac*)).ti,ab.
32	(emergency adj3 (care or setting? or facilit* or ward? or department? or service?)).ti,ab.
33	(after hour? or afterhour? or "out of hour?" or ooh).ti,ab.
34	(clinic? or visit?).ti,ab.
35	((health* or medical or walk-in or walkin) adj2 (center? or centre?)).ti,ab.
36	(first contact or "first point of contact").ti,ab.
37	(general practi* or primary care or primary health* or family pract* or family physician?).in,jw.
38	20 or 21 or 22 or 23 or 24 or 25 or 26 or 27 or 28 or 29 or 30 or 31 or 32 or 33 or 34 or 35 or 36 or 37
39	19 and 38
40	1 or 39
41	(uninsured or "no insurance" or "lack of insurance" or medicare or Medicaid or "affordable care act").ti,ab.
42	(safety net adj3 (hospital? or clinic? or program* or provider* or system? or center? or institute*)).ti,ab.
43	(US adj2 safety net).ti,ab.
44	41 or 42 or 43
45	40 not 44
46	((gene* adj3 (risk or screen*)) or screening or proband).ti,ab.
47	45 not 46
48	Terminal Care/ or Terminally Ill/ or (terminal* ill* or terminal care or "end of life care" or palliative care).ti.
49	47 not 48
50	limit 49 to yr="1987 -Current"
51	("31468890" or "31439453" or "31354078" or "30376908" or "21249820" or "27441326" or "30429203" or "31027482" or "31515421" or "31468848" or "30728187").ui.
52	50 and 51
