# Supplementary material for: Optimising GPs’ communication of advice to facilitate patients’ self-care and prompt follow-up when the diagnosis is uncertain: a realist review of ‘safety-netting’ in primary care
Source: BMJ Qual Saf. 2022 Mar 30;31(7):541–54. doi: 10.1136/bmjqs-2021-014529 (PMC9234415; doi:10.1136/bmjqs-2021-014529)
Supplement: Supplementary data [file bmjqs-2021-014529supp005.pdf]

Appendix 5. Included papers

First author (date)	Title	Country	Source type	Participant type	Setting category	Setting	Study design (Disease area)	Number of participants	Is the paper relevant to the question?	Are the methods used appropriate	Are the findings plausible?	Do the conclusions of the study support those of other studies?
**Ackermann (2016)**	Discharge Communication in Patients Presenting to the Emergency Department With Chest Pain: Defining the Ideal Content	Switzerland	Journal article	Physicians and patients	Specialist/ secondary	Hospital	Mixed (Chest pain)	47 ED physicians 51 patients with chest pain	Highly - covers what needs to be communicated on discharge from ED and how.	Yes - data is collected from physicians and patients and combined to reach conclusions, qualitative methods are also used to gain deeper insight into findings.	Yes - the findings are based on appropriate methods and reflect what has been reported elsewhere	Yes
**Akanuwe (2020)**	Communicating cancer risk in the primary care consultation when using a cancer risk assessment tool: Qualitative study with service users and practitioners	UK	Journal article	GPs, practice nurses and patients	In hours	Primary care	Qualitative (Cancer)	17 GPs and practice nurses 19 patients	Moderately - the paper looks at cancer risk communication specifically when a tool (Qcancer) has been used.	Yes - data is collected from multiple perspectives and using qualitative methods which allow in depth exploration of viewpoints. Interview schedules were based on a theoretical framework.	Yes - themes come from the perspectives of multiple participants and data to support them are provided.	Yes

Alam (2017)	Managing diagnostic uncertainty in primary care: a systematic critical review	UK	Journal article	Clinicians, mix of primary and secondary care	Mix	Mix of primary and secondary care	Systematic review<br><br>(All)	Mix - total = 2434	Highly - paper covers the management of diagnostic uncertainty	Yes	To a degree - the review includes a relatively small number of studies (10) and the authors do not rate the quality of the included studies particularly highly.	Yes and no - the authors do not arrive at any unifying conclusions, but the findings of the individual studies do support each other and the literature.
Alaszewski (2005)	Risk communication: identifying the importance of social context	UK	Editorial /letter to editor	NA	Government level communication	NA	Editorial<br><br>(All)	NA	Moderately - discusses risk communication generally	Yes - this is an editorial	Yes - sums up the findings in the papers in recent issues of the journal	Yes - individuals are not passive recipients of information is discussed elsewhere.
Almond (2009)	Diagnostic safety-netting	UK	Commentary	NA	In hours	NA	Commentary/opinion piece<br><br>(All)	NA	Highly - paper discusses safety-netting in first contact situations	Yes - essay is mainly based on an earlier Delphi study conducted by the authors	To a degree - there are no new findings as such presented, rather the findings of the previous Delphi. This study did not involve patient participants so the points on where safety-netting goes wrong are purely from the clinician's perspective. These findings are similar to	Yes.

											those reported elsewhere though	
**Almond (2009b)**	DIAGNOSIS IN GENERAL PRACTICE: Test of time	UK	Commentary	NA	In hours	Primary care	Commentary/opinion piece<br><br>(All)	NA	Highly - paper discusses safety-netting and how it functions	Yes - although this is an essay/literature review	Yes - the findings come from published studies with some analysis from the authors	Yes
**Aronson (2020)**	Development of an App to Facilitate Communication and Shared Decision making With Parents of Febrile Infants ≤ 60 Days Old	USA	Journal article	Parents and clinicians	Specialist/ secondary	Urban quaternary-care academic medical centre with a paediatric ED	Mixed<br><br>(Fever)	Interviews: 27 parents, 23 clinicians App testing: 6 parents, 2 physicians, 1 nurse	Moderately - covers some ways of communicating and parents' information needs but is primarily around lumbar puncture	yes	Yes	Yes - the conclusions/findings around communication support those of other studies.
**Ashdown (2016)**	Prescribing antibiotics to ‘at-risk’ children with influenza-like illness in primary care:	UK	Journal article	GPs	In hours	Primary care	Qualitative<br><br>(Acute respiratory illness)	41	Moderately - contains some information on how patient characteristics influence GP	Yes	Yes	Yes - similar influences mentioned

	qualitative study								decision making			
**Balla (2012)**	Clinical decision making in a high-risk primary care environment: a qualitative study in the UK	UK	Journal article	GPs	Urgent. OOH, walk in	Out of hours primary care	Qualitative<br><br>(All)	21	Moderately - GPs discuss how they deal with patients in a setting with high levels of uncertainty	Yes	Yes	Only a small part of this study was relevant and this is not part of the conclusions. The parts that are relevant to support other studies.
**Bankhead (2011)**	Safety netting to improve early cancer diagnosis in primary care: development of consensus guidelines.	UK	Report	Primary care cancer experts, GPs, GP cancer leads	In hours	Primary care	Mixed<br><br>(Cancer)	54	Highly - covers what should be included in safety-netting and how it should be delivered including for those with language and literacy barriers to communication. Is only from the perspective of the clinician though.	Yes but lacks patient input	Yes	Yes
**Barratt (2018)**	Nurse practitioner consultations in primary health care: patient, carer, and nurse	UK	Journal article	Patients, carers, nurse practitioners	In hours	Primary care	Qualitative<br><br>(All)	9 patients, 2 carers, 3 nurse practitioners	Highly - covers communication and how/why it is effective including from the patients' perspective	Yes - only reservation is over the small numbers of participants in each category. Only 3 nurses and 2 carers.	Yes - they seem to be but again the small number of participants in some categories is a limitation	Yes

	practitioner qualitative interpretations of communication processes											
**Bertheloot (2016)**	How do general practitioners use 'safety netting' in acutely ill children?	Belgium	Journal article	GPs	In hours	Primary care	Qualitative<br><br>(All)	37	Highly - covers how GPs safety-net children but only from the perspective of the clinician	Yes but lacks patient input	Yes	Yes - for the most part but GPs also say that they have no need for SN guidance or support which has not come up elsewhere
**Bhise (2018)**	Patient perspectives on how physicians communicate diagnostic uncertainty: An experimental vignette study	USA	Journal article	Parents	Specialist/ secondary	Paediatric academic centre	Cross-sectional<br><br>(Stomach pain)	71	Moderately - sheds light on how uncertainty communication styles can change perceptions of clinician trustworthiness and competence	Yes - groups are randomised but could have had a larger sample (25, 25, 21 per group)	Yes - implicit communication of uncertainty scores better on trust, competence, and confidence	Yes
**Birt (2014)**	Responding to symptoms suggestive of lung cancer: a qualitative interview study	UK	Journal article	Patients	Specialist/ secondary	Specialist respiratory clinics	Qualitative<br><br>(Cancer)	35	Highly - covers help seeking from the patient's perspective	Yes	Yes	Yes

Black (2015)	Patients' Experiences of Cancer Diagnosis as a Result of an Emergency Presentation: A Qualitative Study	UK	Journal article	Patients	Urgent. OOH, walk in	Emergency departments	Qualitative (Cancer)	27	Moderately - covers how patients make the decision to reattend and follow-up	Yes	Yes - emphasises the multiple attendances often to different settings of patients diagnosed as an emergency	Yes
Boase (2012)	Tinkering and tailoring individual consultations: how practice nurses try to make cardiovascular risk communication meaningful	UK	Journal article	Primary care nurses	In hours	Primary care	Qualitative (Cardiovascular disease)	28	Moderately - discusses CVD risk communication with patients in general rather than safety-netting specifically	Yes	Yes - presents the way nurses try to make information useful for patients in varying ways which are plausible	Yes - again the importance of tailored information is highlighted.

**Brach (2012)**	Ten Attributes of Health Literate Health Care Organizations	USA	Commentary	NA	Mix	Health care organisations in general	Commentary/opinion piece<br><br>(All)	NA	Slightly - focus is on health literacy which is relevant but only a small part of safety-netting. Also is a US paper so contains information about payment for health care which is not relevant.	Unclear - the methods used are not described. This appears to be a literature review and if this is the case a systematic review would arguably have been better.	Yes - contains a lot of plausible and common sense information about health literacy and supporting health literacy.	Yes - much of the points raised are common sense and support other studies.

Buntinx (2011)	Dealing with low-incidence serious diseases in general practice.	Netherlands, UK, Germany (authors)	Commentary	NA	In hours	Primary care	Commentary/opinion piece<br><br>(All)	NA	Moderately - discusses a range of ways that low incidence serious disease should be catered for in primary care, including a section on safety-netting.	This is a discussion piece so uses a literature review. A systematic review would have been more robust but this is appropriate as a starting point.	There are not any findings as such - presents some from other papers but is mainly raising questions and the gaps in the literature.	Yes - the conclusions are mainly a call for further research but the suggestions are appropriate.

**Byrne (2016)**	'Brief, Superficial' ED Interactions Spur Litigation	USA	Web page	NA	Urgent. OOH, walk in	Emergency departments	Web magazine opinion piece<br><br>(All)	NA	Moderately - describes the issues that can arise with poor communication, including of uncertainty. Gives some strategies to improve communication.	This is an expert opinion piece which is not inappropriate but could be better researched using different methods.	Yes - the advice given is fairly standard good communication advice	Yes - the advice given is fairly standard good communication advice
**Cabral (2014)**	''They just say everything's a virus''—Parent's judgment of the credibility of clinician communication in primary care consultations for respiratory tract infections in children: A qualitative study	UK	Journal article	Parents	In hours	Primary care	Qualitative<br><br>(Respiratory tract infection)	30	Highly - parents are discussing what makes clinician communication credible in an illness area where safety-netting is likely to be frequently used	Yes	Yes - parents' expectations of the consultation and their concerns shape the credibility of care recommendations, as well as the effectiveness of clinician communication	Yes - the expectations and concerns of the patient can be a barrier to the acceptance of the clinician's advice and patients value specific and practical advice.
**Campion-Smith (2017)**	Primary care: 10 Top Tips	UK	Web page	NA	In hours	Primary care	Web page<br><br>(Cancer)	NA	Highly - directly discusses ways to improve safety-netting	Unclear - this is a webpage of tips for safety-netting, methods are not detailed but it is likely expert opinion.	Yes - author and Macmillan who produced the piece are trustworthy.	Yes - recommendations/tips are similar to those in other literature.

Cancer Research UK (2020)	Safety netting patients during the COVID-19 pandemic	UK	Web page	NA	In hours	Primary care	Web page<br><br>(Cancer)	NA	Highly - directly discusses safety-netting	Unclear - no discussion of methods	Yes - good source and plausible recommendatio ns	Yes - recommendations are supported by other literature
Cancer Research UK (2020)	Safety netting: Coronavirus (COVID-19)	UK	Web page	NA	In hours	Primary care	Web page<br><br>(Cancer)	NA	Highly - CRUK summarises the advice on safety-netting	Yes - it is a basic literature review so not comprehensive but the information is based on the main papers on the topic.	Yes - based on the main papers on the topic.	Yes.
Carter (2020)	A systematic review of the organizationa l, environment al, professional and child and family factors influencing the timing of admission to hospital for children with serious infectious illness	UK	Journal article	Parents of child patients and first contact health care profession als	Mix	Primary, urgent, and emergenc y care in high income countries	Systematic review<br><br>(Serious infectious illness)	12 papers 6380 papers	Moderately - discusses the factors that can lead to delayed presentations of serious illness in children so useful for things safety-netting should try to address	Yes - aim of the paper is well met by SR method	Yes - the factors described are plausible particularly in relation to parent and clinician factors. The authors note weak evidence around the organisational factors reported.	Yes - the authors suggest how parents and clinicians can be supported to avoid delays that are in line with other research.

Chen (2019)	Improving assessment of children with suspected respiratory tract infection in general practice	UK	Journal article	GPs	In hours	Primary care	Trial of an EMIS template following Plan Do Study Act cycles<br><br>(Respiratory tract infection)	NS (1 practice)	Slightly - paper mainly discusses the development of the tool, relevant part are around what information should be given and how safety-netting should be recorded.	Yes - methods seem appropriate but study is limited to 1 practice.	Yes - the study was iterative and took feedback into account at each stage to form the results.	Yes - that a template will improve documentation.
Colliers (2020)	Understandin g General Practitioners' Antibiotic Prescribing Decisions in Out-of-Hours Primary Care: A Video-Elicitation Interview Study	Belgium	Journal article	GPs	Urgent. OOH, walk in	Out of hours primary care	Qualitative<br><br>(Bacterial and viral infections)	21	Moderately - paper has a section on safety-netting in OOH which is relevant	Yes - recorded consultations and interviews using the consultations as prompts	Yes - describes the SN behaviours of GPs which are similar to those reported previously.	Yes - paper fits in with the rest of the literature.
Conti (2016)	Informal caregivers' needs on discharge from the spinal cord unit: analysis of perceptions and lived experiences	Italy	Journal article	Carers	Communit y	Communit y	Qualitative<br><br>(Spinal cord injury)	11	Slightly - gathers the views of carers of adults with spinal injuries but mainly on their changing identity and coping strategies. Only a small part on information	Yes	Yes - describes the impact and adjustment of the carer to their new caring role plausibly	Yes - but with limited usefulness for this study.

									needs.			
de Bont (2014)	A separate 'traffic light' for every child with a fever?	The Netherlands	Comme ntary	NA	Mix	Primary and secondary care	NA<br><br>(Fever)	NA	Moderately - cover how the need for information can affect how a parent consults and briefly how SN can help.	NA	Yes - discusses a traffic light tool to help gauge how serious the child's illness is.	Yes - from an evidence based guideline.
de Vos-Kerkhof (2016)	Tools for 'safety netting' in common paediatric illnesses: a systematic review in emergency care	The Netherlands	Journal article	Child patients	Urgent. OOH, walk in	Emergenc y departme nts	Systematic review<br><br>(All)	58 articles median 1371 children	Moderately - Discusses the evidence for safety-netting strategies for children attending ED but only very broadly, is more concerned with whether safety-netting can reduce ED reattendance.	Moderately - the use of a systematic review is appropriate but the range of ages included is large (one study has an upper quartile of 21 years) and results are generalised across ages. Breaking down by age groups may have been better.	Yes - mostly discusses the conflicting evidence for safety-netting preventing reattendance.	Moderately - the conclusions around the characteristics of children that lead to reattendance are in line with other studies. On safety-netting the authors suggest that a high-risk group in need of safety-netting advice is hard to identify, whereas much of the literature suggests that safety-netting should be done consistently.

Dekker (2018)	Parents' attitudes and views regarding antibiotics in the management of respiratory tract infections in children: a qualitative study of the influence of an information booklet	The Netherlands	Journal article	Parents of child patients	In hours	Primary care	Qualitative<br><br>(Respiratory tract infections)	18	Moderately - specifically relevant to the information needs of parents consulting with children but also covers attitudes to antibiotic prescribing which is less relevant.	Yes	Yes - support provided to parents in the form of a leaflet made them more confident about a watch and wait approach and the particularly valued information on when to re-contact the GP.	Yes - other studies have reported that parents value information on when to come back to the GP and the support that additional information materials provide.
Derkx (2008)	Quality of clinical aspects of call handling at Dutch out of hours centres: cross sectional national study	Netherlands	Journal article	Triagists at out of hours call centres	Urgent. OOH, walk in	Out of hours call centres	Cross-sectional<br><br>(All)	17 out of hours centres, 357 calls	Moderately - a small amount of discussion about the quality of safety-netting advice given in this setting.	Yes - calls were made to call centres with standardised patient scenarios and the type and quality of advice and triage were measured.	Yes - a good number of calls were made and the findings are across patient scenarios.	Moderately - the quality of safety-netting advice is rarely measured but these conclusions are supportive of the literature.

Driedger (2013)	Communicating Risk to Aboriginal Peoples: First Nations and Metis Responses to H1N1 Risk Messages	Canada	Journal article	First nations and Metis adults	Community	Community	Qualitative (Pandemic flu)	20 interviewees 193 in focus groups	Moderately - it discusses health risk communication during a pandemic with people from an ethnic minority. Although these ethnic groups are not common in the UK the paper discusses useful points around communicating risk with underserved populations and groups who may a different view or mistrust of the health care system.	Yes - key informants and community leaders were used to recruit participants and a qualitative method allows for the views of the participants to be explored in depth.	Yes - they recruited a large sample of men and women and presented plausible findings with supporting quotes.	Yes - although this study is in a very specific ethnic group, the findings are supported by those involving other groups.
Edwards (2004)	Patient-based outcome results from a cluster randomized trial of shared decision making skill development	UK	Journal article	GPs	In hours	Primary care	Randomised trial (Non-valvular atrial fibrillation; prostatism; menorrhagia; and menopause-	20	Moderately - specifically relevant to the process of negotiating what is decided in the consultation.	Unclear - the methods section does not have a lot of detail and references an online appendix that is not available.	Yes - although the study found no effect of the intervention, the provision of more time and a protected environment for consultations improved confidence and	Yes - that shared decision making and risk communication is important

	and use of risk communication aids in general practice.						related problems)				expectations of adhering to the GP's advice.	
Engel (2012)	Patient understanding of emergency department discharge instructions: where are knowledge deficits greatest?	USA	Journal article	Adult patients	Urgent. OOH, walk in	Emergency departments	Cohort<br><br>(Ankle sprain, back pain, head injury, kidney stone, and laceration)	159	Highly - discusses the self-care information needs and deficits of patients after discharge including dealing with lower health literacy and reading skills	Yes - patients were interviewed 24-36 hours after discharge about whether they could recall and understand key pieces of information and recollections were compared with patient notes.	Yes - knowledge deficits were common and more likely in home care and return to ED instructions. Deficits were reduced when the patient had read their discharge instructions.	Yes - supports that patients can find it difficult to retain all information on discharge particularly when it is not related to the diagnosis itself and that take-away information can help this.
Evans (2018)	GPs' understanding and practice of safety netting for potential cancer presentations: a qualitative study in primary care	UK	Journal article	GPs	In hours	Primary care	Qualitative<br><br>(Cancer)	25	Highly - discusses the practice of SN with UK GPs	Yes - in depth interviews are appropriate for gathering information about personal experiences and understanding	Yes - the findings summarize the views of a number of GPs	Yes - supported by previous research and subsequent research.

**Evans (2019)**	How do GPs and patients share the responsibility for cancer safety netting follow-up actions? A qualitative interview study of GPs and patients in Oxfordshire, UK	UK	Journal article	GPs and adult patients	In hours	Primary care	Qualitative<br><br>(Cancer)	25 GPs<br>23 patients	Highly - discusses safety-netting specifically and particularly follow-up	Yes - qualitative allows participants to discuss their views more deeply	Yes - responsibility for follow is generally shared and moves from GP to patient depending on needs	Yes
**Filler (2020)**	Multi-level strategies to tailor patient-centred care for women: qualitative interviews with clinicians	Canada	Journal article	Clinicians, mix of primary and secondary care	Mix	Primary and secondary care	Qualitative<br><br>(Cardiovascular disease, depression, contraception)	37	Moderately - does not discuss safety-netting specifically but discusses how communication can be facilitated between the clinician and female patient.	Yes - the authors state that the views of clinicians in this area are uncommon so a qualitative method for an exploratory study is appropriate.	Yes - the findings discuss building trust, dealing with uncertainty, and tailoring information to the patient context so are plausible.	Yes - the findings are supported by and support other studies.
**Frame (2016)**	Safety Netting	UK	Web page	NA	In hours	Primary care	Web page<br><br>(All)	NA	Moderately - from a medico legal perspective	Unclear - this is a commentary piece and very few references are cited. The recommendations are in line with what is usually recommended regarding safety-netting though.	Yes - most of the commentary mirrors what is often discussed. The information given from the risk management perspective seems plausible	Yes

											since the author is a risk advisor at MDDUS but his claims are not supported by evidence.	
Gol (2019)	Symptom management for medically unexplained symptoms in primary care: a qualitative study	Netherlands	Journal article	GPs and adult patients	In hours	Primary care	Cross-sectional<br><br>(Medically unexplained symptoms)	18 GPs<br>39 Patients<br>39 consultations	Moderately - the uncertainty involved in medically unexplained symptoms and the strategies to manage that uncertainty is useful here.	Yes - coding was also carried out by a diverse team in terms of their gender and specialties	Yes - themes reported are plausible.	Yes - the different strategies for managing the uncertainty and the shortfalls in advice given have been more widely reported.
Gray (2018)	Augmenting the safety netting process & reducing unnecessary re-presentations	UK	Web page	Health-care professionals (HCPs)<br>Parents and carers attending PAU<br>Parents and carers re-presenting to PAU	Specialist/ secondary	Hospital paediatric assessment units	Web page<br><br>(All)	NS	Highly - improvements that could be made to the safety-netting process were explored and the changes made. Changes in unnecessary re-presentations are reported. Considers communication strategies	Unclear - this is a short summary of the work and little detail is given on the methods. From the information that is given, the methods seem to be appropriate.	Moderately - little detail is given on the findings of the different stages of the work. The main finding seems to be that if patients are given written information to take with them they are less likely to represent unnecessarily	Yes - written information is recommended often so yes.

									for those with low literacy.		which is plausible.	
Gray (2019)	Fifteen-minute consultation: Safety netting effectively	UK	Commentary	Parents/ child patient	In hours	Primary care	Commentary/opinion piece<br><br>(All)	NA	Highly - paper discusses the way safety-netting of child patients should be done and the common pitfalls	Unclear - this is a commentary piece and the authors have drawn on a range of studies, it is possible though that it could have been made more robust of a systematic review was done.	Yes - all the recommendations are common sense and based on cited evidence.	Yes - conclusions are supported by the cited research and are in line with what is considered to be how safety-netting should be done.
Greenhalgh (2020)	Safety netting; best practice in the face of uncertainty	UK	Commentary	NA	Specialist/ secondary	Musculoskeletal physiotherapy	Commentary/opinion piece<br><br>(Musculoskeletal issues)	NA	Highly - discusses what should be included in safety-netting advice in a non-GP setting	Unclear - this is a literature review where the majority of the cited work will have been conducted in general practice so there may be something relevant to physiotherapy that is not captured.	Yes - it is plausible that the content of safety-netting in physiotherapy will be similar to that in general practice.	Yes - GP safety-netting literature supports this.

Gustafsson (2018)	Need for reassurance in self-care of minor illnesses	Sweden	Journal article	Adult patients	Communit y	Communit y	Qualitative<br><br>(“Minor” illness)	12	Highly - investigates how patients can be helped to care for themselves and what their specific needs are.	Yes - Patients were recruited through the Swedish Healthcare Direct which is similar to NHS 111 and efforts were made to get a diverse sample. Qualitative methods are appropriate to explore patients' needs. The sample size is fairly small for a qualitative study.	Yes - reassurance, self-care information, and feeling able to return were among the factors highlighted as important.	Yes - the needs and concerns of these patients echo those reported previously.
Halls (2017)	Qualitative interview study of parents’ perspectives, concerns and experiences of the management of lower respiratory tract infections in children in primary care	UK	Journal article	Parents of child patients	In hours	Primary care	Qualitative<br><br>(Lower respiratory tract infections)	25	Moderately - has some findings around the information that parents need when caring for a child with chest infection but a lot of the paper is also about understanding antibiotics/ antimicrobial resistance	Yes - parents were recruited through practices based on having attended with their child suffering from a LRTI. Interviews were done face to face. Only reservation is how memorable the circumstances were if the parent was interviewed over 2 years previously.	Yes - LRTI can be concerning for parents and they expect symptomatic relief from the GP. Parents often did not recall advice given about infection duration and natural history.	Yes - findings support those of other studies looking at LRTI and other disease areas.

**Healthy London Partnership 2016**	Pan-London suspected cancer safety netting guide	UK	Web page	ALL	Mix	Primary care and dentists	Web page<br><br>(Cancer)	NA	Highly - provides aspects that should be included in safety-netting but nothing about why although this is not unusual.	Yes - the tips were gathered from resources put out but bodies like NICE and CRUK so for the type of resource this is it is appropriate.	No findings as such but the tips are from good sources	Yes - the tips are taken from other sources and are in line with the wider literature
**Henry (2018)**	Canadian Pandemic Influenza Preparedness : Communications strategy	Canada	Journal article	NA	Government level communication	All health communication settings	NA<br><br>(Pandemic flu)	NA	Moderately - discusses risk communication during a pandemic	Unclear - article summarises a report but how the report was created is not discussed.	Yes - the article is the result of a government report which should be a trustworthy source	Yes - stresses the need for information to avoid anxiety and misinformation which has support in the literature.
**Heyhoe (2020)**	The early diagnosis of cancer in primary care: A qualitative exploration of the patient's role and acceptable safety-netting strategies.	UK	Journal article	GPs and adult patients	In hours	Primary care	Qualitative<br><br>(Cancer)	10 HCPs<br>5 patients<br>18 stakeholders	Highly - gathers views on and assesses 3 safety-netting strategies (verbal discussion and plan, written information, prompt)	Yes - interviews are appropriate for understanding something complex although 5 patient interviews is a low number. Workshopping is appropriate for finding the pros and cons of different strategies.	Yes - from a range of sources	Yes - the themes around safety-netting are similar to what has been reported and the stakeholders' concerns about prompts are supported and support previous findings.
**Hirst (2018)**	Acceptability of text messages for safety netting patients with low-risk cancer symptoms: a qualitative	UK	Journal article	GPs	In hours	Primary care	Qualitative<br><br>(Cancer)	22	Moderately - discusses a different way to safety-net patients using text messages	Yes - assessing the feasibility of text message safety-netting through discussion	Yes - text messaging was not recommended in all circumstances but where it was is plausible	Yes - not the specific findings about text messaging as a communication tool but around the need for additional and reinforced information

	study											
**Holmes (2008)**	Communicating about emerging infectious disease: The importance of research	Canada	Journal article	All	Government level communication	All health communication settings	Literature review (Emerging infectious disease)	NA	Moderately - discusses the role of assessing risk and trust in the patient accepting information which is relevant.	Unclear - this is a literature review, it is unclear why a systematic review was not done.	Yes - findings are broad to infectious disease generally but plausible.	Yes - supported elsewhere
**Holmes (2009)**	Communicating with the public during health crises: experts' experiences and opinions	Canada	Journal article	Public health officials, scientists, and communications professionals	Government level communication	Local government and health authorities	Qualitative (Health crisis/ pandemic disease)	22	Moderately - although it doesn't discuss safety-netting it does discuss risk communication during a health crisis which is relevant to part of our question.	Yes - qualitative interviews	Yes - the factors of effective communication reported are plausible	Yes - the conclusions highlight the importance trust, knowing what needs to be achieved, and two-way communication which are concepts that are supported in the literature.
**Houston (2000)**	'Do I don't I call the doctor': a qualitative study of parental perceptions of calling the GP out-of-hours.	UK	Journal article	Parents of child patients	Urgent. OOH, walk in	Out of hours primary care	Qualitative (All)	29 families	Moderately - discusses the factors that prevent a parent caring for their child in the wider context of OOH use.	Yes - In depth interviews are appropriate for gathering perceptions	Yes - discusses the illness and psychological factors at play in parents' decisions to consult OOH.	Yes - knowledge and confidence are important in self-care

Jackson (2005)	Communication About Symptoms in Primary Care: Impact on Patient Outcomes	USA	Journal article	Adult patients (excluding those presenting with URTI symptoms) Clinicians	Urgent. OOH, walk in	Walk in clinic Walter Reed Army Medical Centre	Cohort (All)	Patients: 500 Clinicians: 28	Moderately - discusses communication during the consultation and satisfaction, worries, and unmet expectations with it.	Yes - the study explored correlations between what was communicated in the consultation from the perspective of the patient and the clinician and the resulting satisfaction with the consultation using validated tools.	Yes - agreement between patients and clinicians was high on concrete aspects of the consultation such as prescribing but lower on aspects of communication such as diagnosis or prognosis. When these discussions happened satisfaction with the consultation was higher.	Yes - that good communication is linked to greater satisfaction, less so that good communication impacts outcomes.
Jarvis (2016)	Playing it safe - safety netting advice	UK	Web page	NA	In hours	Primary care	Web page (All)	NA	Moderately - the commentary is more related to the medico legal concerns around safety-netting but this is a relevant aspect to consider.	Unclear - this is a commentary and gives the author's thoughts on the topic. Arguably the topic could have been more comprehensively discussed if a SR was done.	Yes - in line with the research	Yes - concludes with top tips that echo the research.

Jones (2013)	The safety netting behaviour of first contact clinicians: a qualitative study	UK	Journal article	Doctors and nurses	Mix	Primary and secondary care	Qualitative<br><br>(All)	16	Highly - discusses safety-netting in primary and secondary care with doctors and nurses including with patients with ow literacy or ability to understand English.	Yes - qualitative is appropriate to explore behaviour in depth. The study only has 16 participants but has a range of professionals	Yes - findings are corroborated by the range of professionals and supporting quotes are provided	Yes - many of the findings are supported and support other research
Jones (2014)	Information needs of parents for acute childhood illness: determining 'what, how, where and when' of safety netting using a qualitative exploration with parents and clinicians	UK	Journal article	Parents of child patients	Mix	First contact care settings, communit y centres, children's centres and nurseries in the Midlands, UK	Qualitative<br><br>(All)	27	Highly - directly discusses the best content for safety-netting advice including how to present it for recipients with low literacy, language skills, and cultural backgrounds.	Yes - qualitative study that includes the views of parents from a range of backgrounds and clinicians	Yes - many of the recommendatio ns are common to communication research and safety-netting research.	Yes - conclusions are similar to other studies
Jones (2019)	Safety netting for primary care: evidence from a literature review	Conducted in the UK, includes internationa l papers	Journal article	Patients, carers, health care profession als	Mix	Any health care setting	Systematic review<br><br>(All)	NS	Highly - systematic review of the safety-netting literature.	Yes	Yes - findings summarised the literature and echo what has been published since	Yes - that SN is more than communication of uncertainty but should also include follow-up plans and admin details.

Kai (1996)	Parents' difficulties and information needs in coping with acute illness in preschool children: a qualitative study	UK	Journal article	Parents	Mix	Primary care and the communit y	Qualitative (Acute bacterial/ viral illness)	95	Highly - covers parents' information needs with acutely ill children	Yes	Yes - describes parents' information needs, the difficulties in making sense and coping with an ill child as well as the difficulties experienced in doctor communication both of which affect how satisfied the parent is with the consultation	Yes - results that parents want to be able to understand their child's illness and that information should be provided in a variety of formats are supported by other studies, as is the finding that the doctor-patient relationship and the perceived professionalism of the doctor can affect parents' willingness to follow advice.
Kai (1996b)	What worries parents when their preschool children are acutely ill, and why: a qualitative study	UK	Journal article	Parents	Mix	Primary care and the communit y	Qualitative (Acute bacterial/ viral illness)	95	Highly - covers what concerns parents when presenting with an acutely ill child and their information needs	Yes	Yes - feeling lack of control when parents' understanding of the illness is lacking and fear of serious illness is supported by later literature	Yes - clinicians need to understand parents' concerns and try to address them by acknowledging them and providing information.
Lass (2018)	Contact to the out-of-hours service among Danish parents of small children – a qualitative interview study	Denmark	Journal article	Parents of child patients	Urgent. OOH, walk in	Out of hours primary care	Qualitative (All)	9	Moderately - paper does not discuss safety-netting specifically but has information on what causes worry for parents. The Danish health care system is also	Moderately - qualitative methods are appropriate to meet the aims of the study but the sample is limited in terms of number and gender of the participants.	Yes - and are supported by quotes from the parents but a wider breadth of experience may have added value.	Yes - the conclusions fit with previous and subsequent research.

									relatively similar to the UK.			
Lecky (2020)	Optimising management of UTIs in primary care: a qualitative study of patient and GP perspectives to inform the development of an evidence-based, shared decision-making resource	UK	Journal article	Patients and GPs	In hours	Primary care	Qualitative<br><br>(Urinary tract infections)	Patients: 29<br>GPs: 20	Moderately - paper covers safety-netting of UTIs which are common and usually dealt with by the patient although antibiotics may sometimes be needed which is an area that patients need information on.	Yes	Yes and the inclusion of patients as well as GPs provides balance	Yes - shared decision making and tailored information help to empower the patient to self-care
Lemal (2013)	Health risk communication	USA (published)	Book	All	Government level communication	All	NA<br><br>(All)	NA	Moderately - this book explores health risk communication in general and so there are some relevant general concepts	Yes - each book chapter explores a different aspect of risk communication mostly in the form of a literature review	Yes	Yes - the chapters agree with other research

Leslie (2006)	Fear and Coughing in Toronto: SARS and the Uses of Risk.	Canada	Journal article	NA	Governme nt level communic ation	All risk communic ation specificall y around the SARS outbreak in Toronto in 2003	Qualitative (Pandemic flu)	NA	Moderately - discusses risk communicatio n during a pandemic	Yes - analysis of the discourse in the news media	Yes - plausible discussion of the different ways risk was framed	To a degree - the conclusions are applicable during the COVID outbreak but are very narrow.
MacArtney (2017)	Patients' initial steps to cancer diagnosis in Denmark, England and Sweden: what can a qualitative, cross-country comparison of narrative interviews tell us about potentially modifiable factors?	Denmark, UK, Sweden	Journal article	Adult patients diagnosed with cancer	Specialist/ secondary	Secondary care	Qualitative (Cancer)	155	Highly - discusses the experiences of recently diagnosed cancer patients of consulting and getting a diagnosis including safety-netting.	Yes - qualitative good for exploring experiences and the inclusion of different countries with varying cancer survival rates provides interesting comparisons.	Yes - uncertainty about whether and when to reattend was higher in UK and Denmark and clear action plans are needed.	Yes - it has been reported in other papers that clear action plans are important for timely reconsulting.
Maguire (2011)	Which urgent care services do febrile children use and why?	UK	Journal article	Parents of child patients	Urgent. OOH, walk in	Urgent and emergenc y care services	Mixed (Fever)	220 (questionnai re) 29 (interview)	Moderately - discusses experiences in urgent and emergency care services but also discusses safety-netting in these services briefly	Yes - wide range of perspectives gathered with the survey and then in depth information gathered through the interviews.	Yes - high levels of safety-netting advice given and a need and preference for written information to help remember it.	Yes - reinforces the need for clear consistent and symptoms based advice.

**McKelvey (2010)**	The consultation hill: a new model to aid teaching consultation skills.	UK	Commentary	NA	In hours	Primary care	Commentary/opinion piece<br><br>(All)	NA	Highly - specifically discusses safety-netting	Unclear - this is a commentary/opinion piece, it is unclear how the recommendations were arrived at	Yes - seem to be led largely by Neighbour's work	Yes - supported by previous research and subsequent research.
**McKelvie (2019)**	Challenges and strategies for general practitioners diagnosing serious infections in older adults: a UK qualitative interview study	UK	Journal article	GPs	In hours	Primary care	Qualitative<br><br>(Serious infection)	28	Highly - the paper specifically discusses safety-netting	Yes - the aim was to explore in depth so qualitative approach is appropriate.	Yes	Yes - the safety-netting findings are supported by and support many other studies.
**McKinstry (2011)**	Comparison of the accuracy of patients' recall of the content of telephone and face-to-face consultations: an exploratory study.	UK	Journal article	GPs and adult patients	In hours	Primary care	Cohort<br><br>(All)	10 GPs<br>175 patients	Moderately - contains some information useful to improve recall.	Yes - mix of interviews and questionnaires with recordings of the consultation so that aspects of it could be verified.	Yes - patients generally recalled GPs' advice with only minor inaccuracies. GPs seldom used memory aid techniques.	Yes and no - this study disagrees with a similar one conducted in EDs but agrees with others. Anxiety is thought to decrease recall and it may be that the unfamiliar environment and maybe more anxiety provoking reasons for attending in ED harmed recall.
**Mitchell (2012)**	Improving diagnosis of cancer: A toolkit for general practice	UK	Report	NA	In hours	Primary care	NA<br><br>(Cancer)	NA	Highly - report directly discusses safety-netting	Yes - report draws on a range of studies and literature	Yes - findings come from a range of sources	Yes - the conclusions are well supported

Mitchell (2015)	The role of primary care in cancer diagnosis via emergency presentation: qualitative synthesis of significant event reports.	UK	Journal article	NA	In hours	Primary care	Cross-sectional<br><br>(Cancer)	222 cases of emergency presentation	Moderately - briefly discusses the role of safety-netting in preventing emergency presentations	Yes - analysis of serious adverse events	Yes - plausible factors in emergency presentations discussed	Yes - supported by the literature
Morgan (2014)	Starting off in general practice - consultation skill tips for new GP registrars	Australia	Commentary	NA	In hours	Primary care	NA<br><br>(All)	NA	Highly - discusses primary care consultation skills for new GPs including SN	Commentary paper but draws on the published evidence.	Yes - common sense recommendations backed up by the literature.	Yes - they echo other studies.
Neill (2015)	Parent's information seeking in acute childhood illness: what helps and what hinders decision making?	UK	Journal article	Parents of child patients from a range of ethnic backgrounds	Community	Community	Qualitative<br><br>(Acute childhood illness)	27	Moderately - most of the paper is about where parents from varying cultural/ ethnic backgrounds get health information for their child from. There is some discussion of information from health care providers and what information is needed	Yes	Yes - they echo much of what has been reported previously and provide the perspective of less well researched communities.	Yes - the need for take away information, specific and tailored information, and reassurance are highlighted again.

Neill (2016)	Parents' help-seeking behaviours during acute childhood illness at home: A contribution to explanatory theory	UK	Journal article	Parents of child patients	Communit y	Communit y	Qualitative<br><br>(Acute childhood illness)	27	Highly - discusses how parents make decisions when caring for an acutely ill child	Yes- qualitative methods are appropriate to explore behaviours	Yes - they echo much of what has been reported previously and provide the perspective of less well researched communities.	Yes - the conclusions are supported widely
Newcomb (2020)	Building Rapport and Earning the Surgical Patient's Trust in the Era of Social Distancing: Teaching Patient-Centered Communicati on During Video Conference Encounters to Medical Students.	USA	Journal article	Medical students	Specialist/ secondary	Secondary care	Cohort<br><br>(All)	5 students<br>4 observers	Moderately - carried out in secondary care but the information on how to improve communicatio n over video is reevant	Yes - trialling an education session	Yes - the lessons learnt in particular	Yes - supports the few papers on communication over video.
NICE (2016)	Sepsis: recognition, diagnosis and early management	UK	Guidelin e	ALL	Mix	Primary and secondary care	NA<br><br>(Sepsis)	NA	Moderately - contains some advice on safety-netting for potential sepsis but the majority of the guideline is about the diagnosis and management	Yes - NICE guideline using established gold-standard methods	Yes	Yes - the recommendations for what should be included in safety-netting advice are in line with other research.

									of sepsis.			
NICE (2019)	Fever under 5's: assessment and initial management	UK	Guideline	Children under 5	Mix	Primary and secondary care	NA (Fever)	NA	Moderately - contains some advice on safety-netting for children under 5 with fever but the majority of the guideline is about the diagnosis and early management of fever	Yes - NICE guideline using established gold-standard methods	Yes - comprehensive literature search to find and report all relevant literature	Yes - the recommendations for what should be included in safety-netting advice are in line with other research.
Nicholson (2016)	Can safety-netting improve cancer detection in patients with vague symptoms?	UK	Journal article	ALL	In hours	Primary care	Literature review (Cancer)	NA	Highly - discusses safety-netting specifically for vague symptoms.	Yes - this is a literature review but the purpose is to highlight areas that need more evidence and so it is appropriate.	Yes	Yes - although the main conclusions are that there is not a lot of evidence on the topic, which is plausible.
Nicholson (2018)	Responsibility for follow-up during the diagnostic process in primary care: a secondary analysis of	UK	Journal article	Primary care practitioners	In hours	Primary care	Cross-sectional (Cancer)	2879	Highly - discusses safety-netting directly	Yes - large international survey	Yes - findings come from a range on informants and are plausible	Yes - the conclusions are supported

	International Cancer Benchmarking Partnership data											
Noble (2015)	A randomised trial assessing the acceptability and effectiveness of providing generic versus tailored feedback about health risks for a high need primary care sample	Australia	Journal article	Adult patients	In hours	Primary care (Aboriginal Community Controlled Health Services)	Randomised trial<br><br>(Health risks)	87	Moderately - discusses information given to patients generally and whether it should be tailored or generic but this is useful	Yes - patients were randomised to receive either generic or tailored information with the effects of the information measured in an exit survey. The authors managed to get roughly equal numbers of responses in both study arms.	Yes - tailored feedback was seen as more relevant but no more likely to help them improve their health or easier to understand which is perhaps disappointing but the authors offer some explanations.	No - others studies tend to favour tailored feedback/information more than this study.
O'Cathain (2020)	Drivers of 'clinically unnecessary' use of emergency and urgent care: the DEUCE mixed-methods study	UK	Report	Patients considered clinically unnecessary users of services including parents, young adults, people in areas of social deprivation, and	Urgent. OOH, walk in	Emergency and urgent settings	Mixed<br><br>(All)	Interviews: 48<br>Focus groups: 15<br>Survey: 2906	Highly - Has a lot of information about what patients' needs are and how these may not be being met, this then leads to clinically unnecessary consulting. Includes information of	Yes - a range of methods used to answer the question	Yes - the drivers of unnecessary consultations are plausible and have been tested across the studies.	Yes - the drivers found have been reported previously.

				general population					patients with previous traumatic health incidents			
**Pappas (2019)**	Diagnosis and Decision-Making in Telemedicine	UK	Journal article	Secondary care consultants, GPs, nurses, patients, relatives	Mix	Primary and secondary care	Cross-sectional<br><br>(All)	10 consultations, the number of individuals is not stated	Moderately - discusses communication around diagnosis and decision making over video which is broadly relevant but is done with secondary care consultants	Yes - perhaps could have analysed more consultations	Yes - findings around inter professional and HCP-patient communication are plausible	Yes - particularly around the need to not exclude the patient from the conversation
**Rees (2017)**	Patient Safety Incidents Involving Sick Children in Primary Care in England and Wales: A Mixed Methods Analysis	UK	Journal article	Child patients	In hours	Primary care	Cross-sectional<br><br>(All)	2191 incidents	Moderately - study explores the causes of safety incidents including miscommunication which is relevant here.	Yes - analysis of incident reports	Yes - findings are drawn from a large number of incident reports.	Yes - communication between the GP and parents/care givers is a key area and one that has been highlighted previously.

Rising (2019)	Use of Group Concept Mapping to Identify Patient Domains of Uncertainty That Contribute to Emergency Department Use	USA	Journal article	Patients	Urgent. OOH, walk in	Emergency departments	Qualitative<br><br>(All)	34	Moderately - the paper looks at the uncertainty experienced with symptoms not linked to a health condition. It highlights areas where the patient experiences uncertainty but does not offer ways to address it.	Yes - they are fairly unusual but worked well.	Yes - the participants were asked to complete the sentence: 'When experiencing symptoms, people might go to the ED when they feel uncertain about...' and the responses were clustered and refined. The resulting domains are plausible.	Yes - although they are for the ED context, they echo the results of other studies.
Rising (2020)	Development of the Uncertainty Communication Checklist: A Patient-Centred Approach to Patient Discharge From the Emergency Department	USA	Journal article	Patients	Urgent. OOH, walk in	Emergency departments	Qualitative<br><br>(All)	48	Moderately - discusses a checklist of things to discuss with a patient on ED discharge	Yes- the checklist was developed through searching the literature and refined with 2 rounds of patient input.	Yes - the checklist is plausible and has been discussed in 2 rounds of focus groups.	Yes - the checklist items agree with other research.
Roland (2014)	Safety netting in healthcare settings: what it means, and for whom?	UK	Journal article	Child patients	Mix	Primary and secondary care	Literature review<br><br>(All)	NA	Highly - directly discusses the issues around safety-netting	Unclear - this is a best practice review in the form of a literature review. Other more robust methods could have been used/	Yes	Yes - the paper summarises much of the evidence that was available at the time

Royal College of Paediatrics and Child Health et al (2010)	To understand and improve the experience of parents and carers who need advice when a child has a fever (high temperature)	UK	Report	Parents and carers of child patients under 5 years	Urgent. OOH, walk in	Any urgent care setting (GP, walk-in centre, out of hours GP, children's admissions unit, emergency department, ambulance, NHS direct)	Mixed<br><br>(Fever)	220	Highly - discusses parents' decision making when seeking help for a febrile child at a range of primary and emergency care locations.	Yes - a wide range of opinions gathered through a survey and then explored in greater depth with interviews.	Yes - there were high levels of safety-netting advice reported but of varying quality. Parents valued take home information.	Yes - it is often reported that safety-netting advice is given out frequently but that it is not always communicated well. Parents have said previously that they value information they can refer back to.
Silverston (2014)	Effective Safety-Netting In Prescribing Practice	UK	Commentary	NA	In hours	NA	Commentary/opinion piece<br><br>(All)	NA	Highly - paper discusses safety-netting directly	Moderately - a systematic review may have been more robust	Yes - the author covers many aspects of safety-netting and the recommendations fit with other research.	Yes
Silverston (2020)	SAFER: A mnemonic to improve safety-netting advice	UK	Commentary	NA	In hours	Primary care	Commentary/opinion piece<br><br>(All)	NA	Highly - directly discusses how SN advice might be improved	Moderately - this is a commentary piece/literature review from an author who has published other articles about SN.	Yes - the author has published on the topic of SN before and bases his findings on this work and the work of others.	Yes - the elements of the mnemonic reflect what has been published elsewhere.
Silverston (2021)	Safety-Netting in Remote Consulting	UK	Commentary	NA	In hours	Primary care	Commentary/opinion piece<br><br>(All)	NA	Highly - discusses safety-netting in remote consultations	This is a discussion piece and is useful but it is not as rigorous as a piece of research	Yes - the author has published many times on the topic and cites other research.	Yes - supports other studies.

Singh (2016)	A registrar survival guide... follow up and safety netting	UK	Web page	NA	In hours	Primary care	Web page<br><br>(All)	NA	Highly - Discusses how safety-netting should be done in the consultation	This is a discussion piece so does not go into safety-netting in depth, nor does it give advice for different types of presentations. This is fine for a magazine article but should not be used as the only source of information.	Yes - based around the Calgary-Cambridge consultation model.	No conclusions as such given it's a magazine article but the points raised are the same as those in research articles.
van de Maat (2018)	Development and evaluation of a hospital discharge information package to empower parents in caring for a child with a fever	The Netherlands	Journal article	Parents	Urgent. OOH, walk in	Emergency and outpatient departments	Mixed<br><br>(Fever)	Interviews: 22<br>Focus group: 14<br>Survey: 38	Highly - discusses the information seeking and preferences of parents with a febrile child	Yes - the mixed methods approach works well as the authors are exploring a few different questions.	Yes - they are generally in line with previous research.	Yes - the conclusions about information needs and information formats are supported by and support other research.
van Galen (2018)	Telephone consultations	International	Commentary	NA	In hours	Primary care	Commentary/opinion piece<br><br>(All)	NA	Highly - discusses safety-netting over the telephone	Yes - this is not a systematic review but details of the search are provided. Conclusions are based on literature and expert opinion.	Yes - findings are plausible	Yes - the conclusions are generally taken from the literature and where they are not they are in line with other research.

**Van Os (2021)**	Does safety netting for lung cancer symptoms help patients to reconsult appropriately ? A qualitative study	UK	Journal article	GPs and patients	In hours	Primary care	Qualitative<br><br>(Cancer)	3 GPs<br>20 patients	Highly - discusses the effect that safety-netting has on patient behaviour	Yes - Qualitative interviews	Yes - builds on what has been reported before	Yes - supports what has come beofre and builds on it.
**Vaughan (2009)**	Effective health risk communication about pandemic influenza for vulnerable populations.	USA	Journal article	NA	Government level communication	NA	Literature review<br><br>(Pandemic flu)	NA	Moderately - to communication during a health crisis.	Moderately - the authors do not set out to do a systematic review but one may have been more robust.	Yes - the factors affecting the acceptance of public health messaging are presented and are plausible	Yes - the other pandemic studies in this review support the findings and the authors cite several websites that support them.
**Vrdoljak (2020)**	Cancer and coronavirus disease 2019; how do we manage cancer optimally through a public health crisis?	International	Editorial /letter to editor	NA	Mix	Primary and secondary care	Letter to the Editor<br><br>(Cancer)	NA	Moderately - discusses the impact of COVID on patients' reasoning and how the effects of COVID should be mitigated.	Unclear - this is a letter to editor/literature review which is less robust than primary research but at the time of writing was appropriate.	Yes - but anecdotal some of them	Yes - research that has come out since supports this article.
**White (1997)**	Wrapping things up: a qualitative analysis of the closing moments of the medical visit.	Canada	Journal article	GPs and general internal medicine specialists	In hours	Primary care	Cross-sectional<br><br>(All)	50 HCPs<br>550 Patients	Moderately - discusses how consultations are closed which is where safety-netting usually is	Yes - analysis of recorded consultations is appropriate	Yes - discusses the different types of closures, all of which seem plausible	Yes - the relevant parts of the paper are supported in the literature
