# Supplementary material for: Optimising GPs’ communication of advice to facilitate patients’ self-care and prompt follow-up when the diagnosis is uncertain: a realist review of ‘safety-netting’ in primary care
Source: BMJ Qual Saf. 2022 Mar 30;31(7):541–54. doi: 10.1136/bmjqs-2021-014529 (PMC9234415; doi:10.1136/bmjqs-2021-014529)
Supplement: Supplementary data [file bmjqs-2021-014529supp006.pdf]

Appendix 6 CMOCs and supporting excerpts

CMOC	Explanatory credibility	Supporting excerpt
1. When information is personally relevant and tailored to the patient (C), the safety-netting advice is adhered to (O), because the patient has a sense of ownership, relevance, understanding, and credibility of the information they have been provided (M).	High	*Indeed, participants felt that personalizing the risk information could help individual patients to understand the risk information being communicated to them.* (Akanuwe 2020)<br><br>*Participants were significantly more likely to agree that the tailored feedback was 'relevant to me' compared to the generic feedback* (Noble 2015)
2. When the rationale for the management plan is made clear to patients (C), they accept and follow the safety-netting plan (O), because they can understand and question the reasoning behind it (M).	High	*This current study also identified patient frustration resulting from the lack of exploration by the GP about their condition, or the internalisation of the GP thought process, resulting in the patient feeling either reluctant to question the GP's decision or discuss their concerns on a subject that some feel embarrassed about*. (Lecky 2020)<br><br>*Patients preferred active safety-netting strategies as part of a general preference for thoroughness and a logical approach to diagnostic uncertainty surrounding their lung cancer-relevant symptoms. This allowed patients to understand the diagnostic strategy, in turn making the safety-netting advice easier to understand.* (van Os 2021)
3. When clinicians share their uncertainty around diagnoses (C), the patient reattends appropriately (O) because they are not falsely or overly reassured and they are empowered to return (M).	High	*If the diagnosis is uncertain, that uncertainty should be communicated to the patient (or parent/carer) so that they are empowered to reconsult if necessary*. (Almond 2009)<br><br>*If you are not sure of the aetiology, explain this to the patient. This reduces the risk of false reassurance and most patients appreciate the honesty.'* (Jones 2019)
4. When patients' concerns and expectations are addressed in the safety-netting plan (C), patients' acceptance of the safety-netting plan is increased (O), because they believe the clinician is credible, and they feel seen and taken seriously (M).	High	*When patients feel as though they were heard and understood, they have more ownership over the healthcare delivery process* (Byrne 2016)<br><br>*It is also important to understand what patients' thoughts and concerns are around their symptoms in order to avoid a mismatch of doctor and patient agenda*. (Singh 2016)
5. When patients are provided with clarity on when they should be concerned about their signs/symptoms and what to do (C), they are able to adhere to safety-netting advice, and avoid delay if further medical attention is required as well as unnecessary investigations, urgent visits to the GP, OOH and EDs (O), because patients' understand what needs to be done in what circumstances, and their sense of control and confidence in what needs to be done is increased (M).	High	*Parents' anxieties about failing to recognise a serious illness serve as a reminder that what constitutes common knowledge for doctors may not be readily accessible to parents. Information and education that address parents' concerns may empower parents by influencing perceptions of threat posed by an illness and enhancing personal control*. (Kai 1996b)<br><br>*Patients may underestimate the significance of symptoms, hesitate to re-consult, be concerned about wasting the doctor's time, or may be unaware of their responsibility to*

		follow up investigations. (Evans 2018)
6. When patients are provided with a clear, specific, and practical safety-netting plan (C), the patient self-cares effectively and seeks help appropriately (O) because they find the advice useful, their worries and stress are reduced, and they feel empowered to take on their own care (M).	High	*Patients expected and were willing to accept responsibility, as long as they felt they had received sufficient instruction from their GP*. (Evans 2019)<br><br>*“It's the not knowing what it could be—how to tell—that's what panics me, if I was told what to do, shown what to do and how to do it, I would feel I could manage much better” (Parent 6, group 1)* (Kai 1996)
7. When the information is provided in such a way that it is comprehensible (C), the patient adheres to the safety-netting advice (O), because patient understanding is increased (M).	High	*Although parents suggested information should be free of jargon, they were keen that it should not omit important technical information that would facilitate their understanding*. (Kai 1996)<br><br>*understanding of information is central to any safety-netting intervention*. (Roland 2014)
8. When the information is provided in a way that is memorable (C), patient can adhere to safety-netting advice (O) because patient recall is improved (M).	Moderate	*Chunking increases the likelihood that people can reproduce the information they have received* (Ackerman 2016)<br><br>*[way to improve recall] Reducing the volume of information. 2 Reducing delay from presentation to recall. 3 Ordering information according to priority (the first and last pieces of information in a list are best remembered).* (McKinstry 2011)
9. When information is additionally provided to patients in such a way that they can revisit it (C), the patient is supported to follow the safety-netting advice and is able to adhere to the advice (O) because the risk of forgetting is reduced, they share the information, and can spend more time going over and understanding the information (M).	High	*Father of a 3-week-old infant: “I mean, in some cases, like some people understand better by reading it than just hearing it. Some people understand better by hearing it than reading it, so I mean, it could work both ways. It depends on what type of person it is.”* (Aronson 2020)<br><br>*One parent suggested that information about which signs to look for should be provided in writing as it can be difficult to retain spoken information when distressed* (Maguire 2011)
10. When the safety-netting advice is given consistently to all patients by clinicians (C), patients follow safety-netting advice (O), because self-care with the option to return is normalised by the patient and their ability to self-care is facilitated (M).	Moderate	*Health professionals should ensure that safety-netting advice is given to all, and not just to those perceived to be at highest risk of serious illness.* (Maguire 2011)<br><br>*Parents of children with self-limiting illnesses offered safety-netting advice were less likely to use further services. Thus it is essential that safety-netting advice is offered to all parents. The advice should be more specific to the child’s condition, i.e. what symptoms / signs the parent should look for, and what should prompt a return to the health provider.* (RCPCH 2010)
11.<br>a) When the clinician is aware of the patient’s history and addresses the underlying factors that may make the patient less receptive to	High	*Since EPs don’t have long-term relationships with patients, says Byrne, “that makes it even more important that the communication that does happen in the ED is thoughtful, comprehensive, and compassionate.” He suggests that EPs utilize these risk-reducing*

safety-netting advice (C), the patient follows the safety-netting advice (O), because their fear and anxiety is reduced, and the patient regards the safety-netting advice as actionable (M).		*practices: Practicing "the basics" of good communication. This includes making eye contact, acknowledging each person in the room, apologizing for delays, and demonstrating a willingness to listen*. (Byrne 2016)<br><br>*A range of events were associated with parents' loss of trust in HCPs, such as failure to diagnose (especially when followed by serious illness), absence of clinical examination, conflicting information, ineffective treatment, failure to answer questions and always referring on to others*. (Neill 2016)
b) When the clinician shows they are aware of and addresses the specific healthcare concerns the patient may have because of an ongoing pandemic or healthcare crisis when giving safety-netting advice (C), the patient follows the advice (O) because they evaluate the risk to themselves, they regard the advice as more actionable, and their fear or anxiety is reduced (M).	High	*In the current climate, potential oncology patients' minds are now more oriented toward COVID-19 symptoms, meaning that they may downplay rectal or bladder bleeding, a lump in the breast or other signs of cancer that otherwise would lead them immediately to consult their doctor. Anecdotal evidence suggests that patients are starting to fear a COVID-19 diagnosis more than a cancer diagnosis*. (Vrdoljak 2020)<br><br>*Uncertainty during a pandemic can be accompanied by a high demand for information, increased feelings of fear and anxiety, rapid spread of misinformation, and speculation*. (Henry 2018)
12. When the clinician actively compensates for the impeded non-verbal communication during telephone and video consultations (C), the patient's satisfaction with and adherence to the advice is increased (O) because the patient feels reassured that they have been understood and heard, and their confidence in the safety-netting advice is increased (M).	Moderate	*Difficulties in explaining their symptoms over the telephone raised concern about whether the nurse correctly understood the situation. The sense of the nurse taking the time to listen inspired trust that the nurse had fully understood the situation, and thus, participants relied on the assessment and safely embraced the advice. Participants felt reassured when the nurse was professional, calm and factual. Participants described the consultation as reassuring when the nurse was alert and adequately assessed the situation and identified the problem by asking the right questions. Checking for comprehension was reassuring because it gave the participant a feeling that the nurse really wanted to obtain a clear image of the situation*. (Gustafsson 2018)<br><br>*Add simple statements such as "Let me think just a moment" to give the patient confidence your continued focus. A patient's distress may be more difficult to interpret on video; ask direct questions to understand the patient's emotional state*. (Newcomb 2020)
13. When the set up of video consultations is optimised (C), the patient takes the safety-netting advice on board and adheres to SN advice (O) because distractions are removed and the interpretation of facial expressions is facilitated (M).	Low	*Poor lighting and positioning within the screen limited the physician and patient's ability to interpret each other's facial expressions. The "hidden" face was more distracting than if the conversation had been telephonic, hindering patient trust and physician interpretation of the patient's reaction* (Newcomb 2020)
14. When the clinician is not distracted or does not appear to be distracted during a remote consultation (C), the patient takes the safety-netting	Low	*As the physician looked directly into the camera, the patient described the experience as "intimate" and "comforting," as if he was the physician's sole focus.* (Newcomb 2020)

advice on board (O) because they are able to follow the consultation better, and feel they are the sole focus of the clinician (M).		
15. When the safety-netting plan is made through shared discussion and decision making between the clinician and the patient (C), the patient follows the safety-netting plan (O) because they feel taken seriously, ownership of the plan can be negotiated, and the patient understands that the plan can be adapted (M).	High	*Patients stressed how the strategy helped to foster a feeling of being taken seriously. Important aspects for this were the components of shared discussion and decision-making and the inclusion of a pre-determined follow-up for symptom review.* (Heyhoe 2019)<br><br>*Compliance with any management plan (be it lifestyle/health-seeking behaviour modification, following advice or a course of medication) is dependant on the patient having understanding,*<br>*agreement, and a shared ownership with that plan.* (McKelvie 2010)
16. When the clinician checks that the patient understands the safety-netting advice they have been given and that it can be adapted (C), the patient adheres to the safety-netting advice (O), because the patient's confusion and any misunderstandings are reduced (M).	Moderate	*Check the patient fully understands the safety-netting advice provided especially if the appointment is via telephone.* (CRUK 2020)<br><br>*Methods therefore need to be employed to optimise the verbal safety-netting process. Using teach-back methodology, whereby parents repeat back information given to them, may help improve understanding and reduce re-attendance* (Gray 2018)
17. When the clinician explicitly acknowledges the expertise and personal knowledge of the patient (C), the patient reconsults appropriately (O), because they feel empowered and have confidence in their own judgement (M).	Moderate	*Recognising parental expertise [37, 46], empowering parents to contradict clinicians [22], establishing and sustaining trust [26] and creating supportive conditions for parents to be able to seek help from their GP or other services early in their child's illness course and to know when to reconsult if they child's illness progresses [33] has the potential to positively influence the child's journey to hospital*. (Carter 2020)<br><br>*I'm forever saying "If you're worried, I'm worried," to patients, to Mums and Dads. To really underline you know, "You're the world's expert," is the other thing that I'm always forever saying, "You're the world's expert on your child." (GP21, woman, inner-city practice, 5–9 years as a GP)* (Ashdown 2016)
18. When clinicians ensure the rationale for a follow-up plan are made clear to the patient (C), the patient adheres to the safety-netting advice and is followed up in a timely way (O), because the patient's understanding is increased, potential confusion avoided, and the patient is empowered to act (M).	High	*A clear explanation of the follow-up plan, including the underpinning rationale and ongoing uncertainties, is key to enabling patients to re-consult appropriately*. (Evans 2019)<br><br>*Three visits all by the same physician appeared very effective. Each visit was conducted with the same structured format. The most striking feature of these visits was that the physician explicitly told the patient what was going to happen next*. (White 1997)
19. When clinicians and patients explicitly agree on follow-up plans (C), the patient is followed up in an appropriate time frame (O), because each party knows what is expected of them (M).	High	*To minimise misunderstandings about reattendance, the follow-up plan must be explicit about reassessments, and this must be agreed with the patient*. (Almond 2009b)<br><br>*An agreed follow-up or review date is set and possible outcomes/prognosis discussed so*

		the patient can be helped to identify if further help is needed and, if so, how and when to access this. (McKelvey 2010)
20. When the clinician explicitly invites the patient to return, even/including for the same symptoms (C), the patient promptly seeks additional medical advice when needed (O) because they feel they permission to reconsult (M).	High	*The receiving of self-care advice may be perceived as trivialising, like their concerns were not taken seriously and that they were dismissed. Being invited to return created a feeling that the nurse had listened and taken them seriously* (Gustafsson 2018)<br><br>*There were several other examples where patients continued to be concerned about their symptoms, but the recent HCP advice to self-manage presented a barrier to returning for more help. They felt they could not ask for further advice or investigations.* (Black 2015)
21. When the clinician allows sufficient time for the safety-netting advice (C), the safety-netting advice is fully explained, the clinician checks understanding, answers questions, and discusses patient concerns about the safety-netting plan (O), because the clinician does not feel rushed to end the consultation and the patient does not feel rushed to leave the consultation (M).	Moderate	*Moreover, it is necessary to allow enough time for proper 'safety-netting'*. (Bertheloot 2016)<br><br>*When asked about circumstantial influence, GPs recognized that 'safety-netting' is sometimes a bit careless when the waiting room is overcrowded or when the GP is exhausted.* (Bertheloot 2016)
22. When safety-netting advice is documented in sufficient detail in the patient's notes (C), future clinicians caring for the patient are less likely to have misunderstandings/misinterpretations and will care for the patient more appropriately (O), because know what has been done, discussed, and decided so far (M).	High	*Many participants commented about documenting safety net advice in the medical notes and felt it was important from a medico-legal perspective. They also mentioned this could aid continuity of care "I think this is vital for future [physicians] to realise what has been discussed and said, and is important medico legally" and "I try to outline a 'plan' - i.e. what to do if patient returns so other doctors in the surgery know what's going on. This may include advice given to the patient but not by any means always".* (Bankhead 2011)<br><br>*All of us have time away from our practices. When that happens, how are our vulnerable patients not disadvantaged? Think about systems that will support your patients when you're away. Sharing plans with the patient and a colleague will allow the patient to feel secure and supported while ensuring best possible continuity of care.* (Campion-Smith 2017)
